# Physical and Electrochemical Properties of Soluble 3,4-Ethylenedioxythiophene (EDOT)-Based Copolymers Synthesized *via* Direct (Hetero)Arylation Polymerization

**DOI:** 10.3389/fchem.2021.753840

**Published:** 2021-10-29

**Authors:** Qiang Guo, Jincheng Zhang, Xiaoyu Li, Heqi Gong, Shuanghong Wu, Jie Li

**Affiliations:** ^1^ College of Optoelectronic Engineering, Chengdu University of Information Technology, Chengdu, China; ^2^ Southwest University of Science and Technology, Mianyang, China; ^3^ School of Optoelectronic Science and Engineering, University of Electronic Science and Technology of China, Chengdu, China

**Keywords:** direct hetero(arylation) polymerization, conjugated copolymer, electrochemical, structure–property relationship, 3,4-ethylenedioxythiophene (EDOT)

## Abstract

Over the past decades, π-conjugated polymers (CPs) have drawn more and more attention and been essential materials for applications in various organic electronic devices. Thereinto, conjugated polymers based on the 3,4-ethylenedioxythiophene (EDOT) backbone are among the high-performance materials. In order to investigate the structure–property relationships of EDOT-based polymers and further improve their electrochemical properties, a series of organic solvent–soluble EDOT-based alternative copolymers consisting of electron-rich fragments (fluorene **P1**, carbazole **P2**, and 3,4-alkoxythiophene **P3**) or electron-deficient moieties (benzotriazole **P4** and thieno[3,4-*c*]pyrrole-4,6-dione **P5**) were synthesized *via* direct C–H (hetero)arylation polymerization (DHAP) in moderate to excellent yields (60–98%) with medium to high molecular weights (*M*_n_ = 3,100–94,000 Da). Owing to their various electronic and structural properties, different absorption spectra (*λ*_max_ = 476, 380, 558, 563, and 603 nm) as well as different specific capacitances of 70, 68, 75, 51, and 25 F/g with 19, 10, 21, 26, and 69% of capacity retention after 1,000 cycles were observed for **P1–P5**, respectively. After careful study through multiple experimental measurements and theoretical calculation, appropriate electronic characteristics, small molecular conformation differences between different oxidative states, and well-ordered molecular stacking could improve the electrochemical performance of CPs.

## Introduction

With the rapid development in the field of wearable and flexible electronics during the past decades, π-conjugated polymers (CPs) have drawn more and more attention and been essential materials for applications in organic solar cells (OSCs) (Dou et al., 2013), organic field-effect transistors (OFETs) (Olivier et al., 2014), electrochromic devices (ECDs) (Jensen et al., 2015), and electrochemical capacitors (ECs) (Han and Dai, 2019) because of CPs’ excellent electronic, optoelectronic, and mechanical properties. Thereinto, CP-based ECs are considered one of the next-generation alternative energy storage systems, featuring a number of advantages including low cost, lightweight, environmental friendliness, flexibility, fast charge/discharge capability, and relatively high charge storage capacity. Meanwhile, it is widely accepted that the electrode material plays a crucial role in the capacitive performance of a supercapacitor, and lots of efforts have been put toward the development of new and high-performance electrode materials (Meng et al., 2017). Among all kinds of CPs, polyaniline (PANI), polypyrrole (PPy), and polythiophene (PTh) derivatives have been widely investigated as the active electrode materials in ECs with high pseudocapacitance and low cost. It has been reported that conjugated polymers based on the 3,4-ethylenedioxythiophene (EDOT) backbone exhibit excellent redox activity, high conductivity, and fast redox switching speeds (Liu and Reynolds, 2010). In order to further improve their electrochemical properties, nano-structuring, composite, blending, and copolymerization approaches based on EDOT were attempted and performed well (Shown et al., 2015). Particularly, copolymerization would be a valuable and potential method because the resultant new copolymer would combine the positive properties of both monomers. To date, EDOT-based electrode materials were typically fabricated *via* electropolymerization or *in situ* oxidative chemical polymerization on the current collectors. Accordingly, the molecular structure types of polymers synthesized by these two methods are relatively limited, which is not conducive to the study of the structure–property relationship. For example, the A–B alternative copolymer cannot be readily synthesized by classical electropolymerization or oxidative chemical polymerization. Moreover, the products of electropolymerization generally have problems in further processing, large-scale preparation, and limited molecular structure diversity, due to their infusibility and poor solubility in common solvents. And the products of oxidative chemical polymerization also have problems in the metal ion residue and low reactivity for electron-deficient monomers. In other words, to design more appropriate synthetic methods is of crucial importance to the development of high-performance CPs.

Over the past decade, transition metal–catalyzed direct C–H (hetero)arylation polymerization (DHAP) of non-preactivated (hetero)arenes with (hetero)aryl halides is one of the most ideal and effective methods to construct conjugated polymers, avoiding tedious reaction steps and formation of stoichiometric toxic organometallic byproducts in the traditional organometallic couplings (Mercier and Leclerc, 2013). Furthermore, as compared to electropolymerization and oxidative chemical polymerization methods, π-conjugated polymers synthesized *via* DHAP have been widely applied in the field of high-performance organic semiconductors and have shown a variety of merits, such as a well-defined structure, a diversified molecular structure, high reproducibility, large-scale throughput, and good solubility (Wu et al., 2017). Based on the broad applicability and our continuous efforts in constructing CPs *via* DHAP (Guo et al., 2013; Guo et al., 2016), we herein present a new work in that five organic solvent–soluble EDOT-based alternative copolymers consisting of electron-rich fragments (fluorene, carbazole, and 3,4-alkoxythiophene) or electron-deficient moieties (benzotriazole and thieno[3,4-*c*]pyrrole-4,6-dione) were synthesized effectively *via* DHAP in moderate to excellent yields (60–98%) with medium to high molecular weights (*M*_n_ = 3,100–94,000 Da). Although several similar examples of these polymers have been synthesized before, the relationships between the photophysical and electrochemical properties and their structures have not been systematically investigated (Yamazaki et al., 2013; Kuwabara et al., 2014; Kerszulis et al., 2015; Li et al., 2016a; Robitaille et al., 2017). The relationships between CPs’ molecular structures and their electrochemical properties and microscopic packing properties were carefully investigated through cyclic voltammetry (CV), galvanostatic charge–discharge (GCD), atomic force microscopy (AFM), X-ray diffraction (XRD) analysis, and density functional theory (DFT) calculation. The results indicated that all the physical and electrochemical properties of these EDOT-based CPs were predominantly determined by their electronic characteristics, molecular planarity, and rigidity.

## Results and Discussion

**SCHEME 1:**
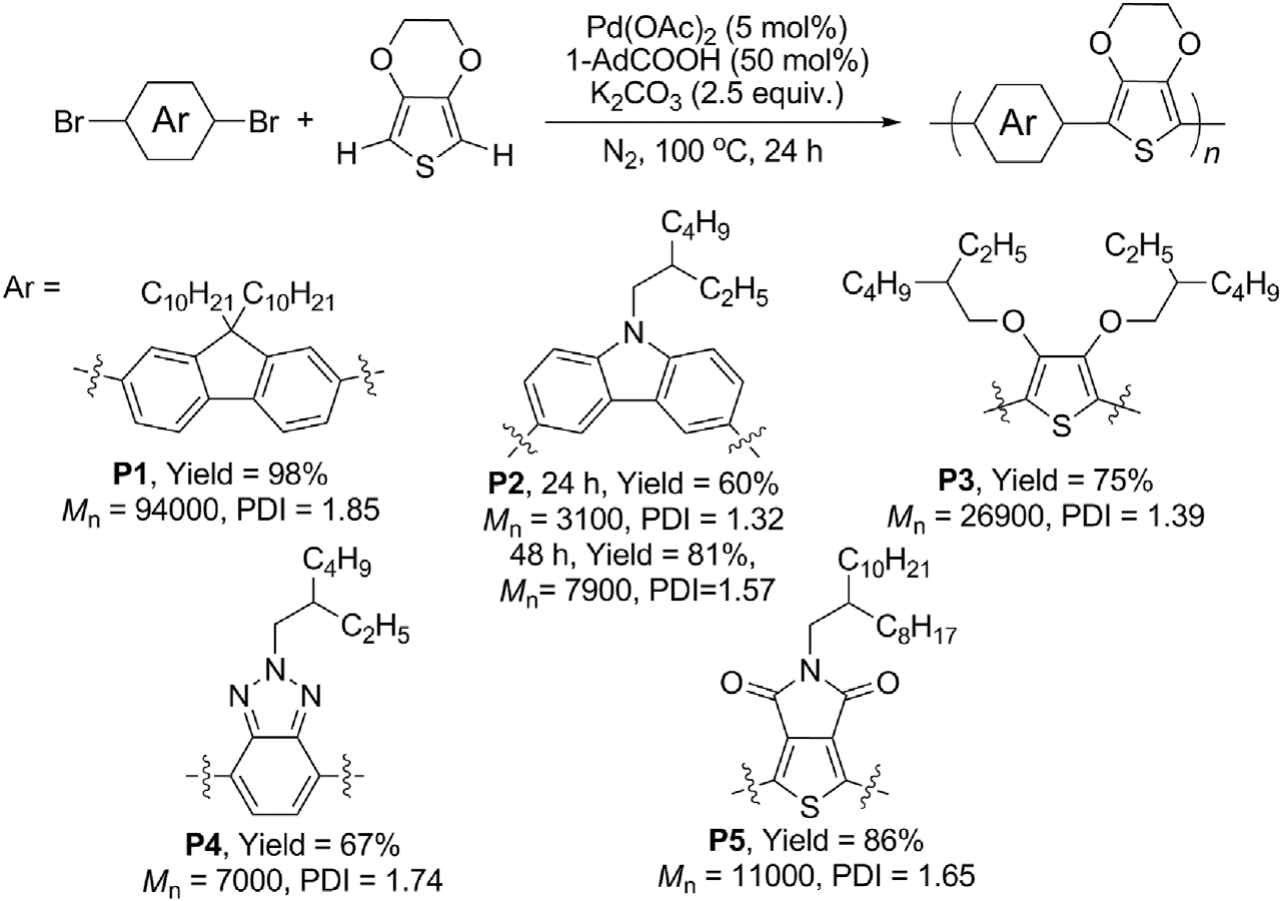
Direct hetero(arylation) polymerization of various EDOT-based CPs.

Owing to the wide application of EDOT-based CPs and high reactivity of C–H bonds, the DHAP of EDOT was early and extensively studied by several research groups (Yamazaki et al., 2013; Elsawy et al., 2015; Hayashi and Koizumi, 2015; Li and Michinobu, 2016). Based on these works, a catalytic system combining Pd(OAc)_2_ (5 mol%), K_2_CO_3_ (2.5 equiv.), and 1-adamantanecarboxylic acid (1-AdCOOH, 50 mol%) in DMAc at 100 C for 24 h was used to synthesize our EDOT-based CPs. As shown in Scheme 1, both electron-rich units (fluorene **P1**, carbazole **P2**, and 3,4-alkoxythiophene **P3**) and electron-deficient units (benzotriazole **P4** and thieno[3,4-*c*]pyrrole-4,6-dione **P5**) were successfully engaged in the reactions, offering structure-diversified EDOT-based CPs in moderate to excellent yields (60–98%) with medium to high molecular weights (*M*_n_ = 3,100–94,000 Da). The yield and molecular weight of **P2** can be further improved up to 81% and 7,900 Da after 48 h, respectively, indicating its relatively low reactivity of 3,6-dibromocarbazole. The good yields of **P1–P5** with comparable or even higher *M*_n_ compared to those obtained in previous works indicated the high reactivity of EDOT under this catalytic system and good applicability of DHAP (Yamazaki et al., 2013; Kerszulis et al., 2015; Li et al., 2016a). It is obvious that the yields and molecular weights of these polymers were closely related to the numbers and lengths of alkyl chains attached on the backbone, namely, good solubility of products can improve the yields and molecular weights. And the relatively low molecular weight of **P4** was probably attributed to its limited solubility because a small amount of insoluble polymer remained in the cartridge after extraction with chloroform. All the polymer structures were confirmed by ^1^H NMR and MALDI-TOF mass spectrometry. The ^1^H NMR spectra of **P1**, **P2**, and **P3** were in agreement with those in the literature (Yamazaki et al., 2013; Kerszulis et al., 2015; Li et al., 2016a). Br terminals can be found in the MALDI-TOF mass spectrum, suggesting the DHAP can be further carried out to provide high-molecular-weight products. Owing to the solubility of **P1–P5** in common solvents (CH_2_Cl_2_, CHCl_3_, and THF), films for characterization of UV-Vis absorption, electrochemical properties, and microscopic packing properties were fabricated by solution processing, such as drip coating and spray coating.

The UV-Vis spectra and data of these five EDOT-based CPs in diluted solution and in films are shown in Supplementary Figure S1 and summarized in Table 1, respectively. Moreover, to gain insight into the structure–photophysical property relationship, the geometries of two repeating unit molecular models for polymers **P1–P5** were optimized through density functional theory (DFT), as shown in Supplementary Figures S15–S19. And the dihedral angles between two units (Table 1) were calculated to measure the coplanarity of polymer molecules. The remarkably different absorption spectra of **P1–P5**, covering the near-UV and blue, green, and red color regions, suggest their distinct electronic and structural properties. The obviously red-shifted absorption of **P1–P5** in the thin film in comparison with that in the solution could be generated from the strong intermolecular interaction between the polymer backbones in film states. On comparison with **P1** and **P2** containing electron-rich units (*λ*_solution_ = 471, 359 nm and *λ*_film_ = 476, 380 nm, respectively), **P4** and **P5** with electron-deficient units displayed significantly red-shifted absorption (*λ*_solution_ = 546, 580 nm and *λ*_film_ = 563, 603 nm, respectively), which should be assigned to the intramolecular charge transfer (ICT) from the EDOT donor to corresponding electron-deficient acceptor units. When connecting 3,4-alkoxythiophene with EDOT, **P3** also displayed a red-shifted absorption (*λ*_solution_ = 547 nm and *λ*_film_ = 558 nm), owing to its almost planar backbone (*θ*_dihedral_ = 2.88^o^) induced by an intramolecular O∙∙∙S interaction (Gleiter et al., 2018). Thus, combining the ICT property and good coplanarity (2.19^o^), **P5** exhibited the most red-shifted maximum absorption wavelength among these five polymers, implying its efficient electron delocalization, transport property, and close molecular stacking.

**TABLE 1:** Physical, electrochemical, and conformational properties of **P1**–**P5**.

Polymer	Experimental data							Calculated data^h^	
	*λ*_solution_ [nm]^a^	*λ*_film_ [nm]^b^	C [F/g]^c^	C_retention_ ^d^ (%)	IR_drop_ [V]^e^	R_a_ [nm]^f^	*T*_d_ [^o^C]^g^	*θ*_dihedral_ [^o^]^i^	RMSD^j^
**P1**	471	476	70	19	0.25	3.91	389	27.15	0.6324
**P2**	359	380	68	10	0.25	4.08	371	29.57	0.6485
**P3**	547	558	75	21	0.08	3.10	316	2.88	0.5437
**P4**	546	563	51	26	0.09	2.91	354	10.81	0.3827
**P5**	580	603	25	69	0.09	2.08	394	2.19	0.1129

a Maximum absorption wavelength in dilute CH_2_Cl_2_ solution.

b Maximum absorption wavelength in the film deposited by spray coating on a quartz plate.

c Specific capacitance at a scan rate of 100 mV s^−1^.

d Capacity retention after 1,000 cycles at a scan rate of 100 mV s^−1^.

e IR_drop_ values measured during the discharge process at 10 A g^−1^.

f Surface average roughness determined by AFM.

g Temperature at 5% weight loss under nitrogen.

h Based on the optimized dimers.

i Dihedral angles between two units.

j Maximum value of RMSD between neutral, 50% doped, and 100% doped states.

Electrochemical measurements were performed in a three-electrode cell with a platinum wire as the counter electrode, an Ag/Ag^+^ wire (silver wire in 0.01 M AgNO_3_ in acetonitrile) as the reference electrode, and a Pt disk or foam-nickel electrode drop-coated with the polymer as the working electrode and using 0.1 M tetrabutylammonium hexafluorophosphate in dry acetonitrile as the electrolyte solution. Firstly, cyclic voltammetry (CV) measurements of **P1–P5** films on a Pt disk electrode were performed to analyze their p-doping/dedoping processes at a scan rate of 100 mV s^−1^ (Supplementary Figure S2). The different CV curves indicated large differences in their electrochemical properties. The corresponding HOMO energy levels were then estimated according to the following equation: HOMO = −(4.80 + *E*_ox,onset_) eV, where *E*_ox,onset_ is the onset potential of the oxidation peak with respect to the ferrocene/ferrocenium (Fc/Fc^+^) redox couple. The HOMO energy levels of **P1** (−5.17 eV), **P2** (−5.06 eV), and **P3** (−4.79 eV) are in good agreement with those reported by other groups (−5.2 eV, −5.09 eV, and ∼ −4.8 eV, respectively) (Kuwabara et al., 2014; Kerszulis et al., 2015; Li et al., 2016a). Compared to the lowest HOMO energy level of **P5** (−5.35 eV) among **P1–P5**, the coplanar and electron-rich **P3** showed the highest HOMO energy level (−4.79 eV), indicating its easiness of losing electrons and the potential development of new polymeric anodes. The arrangement trends of HOMO energy levels of **P1–P5** estimated by CV were in accordance with those of HOMO values calculated by DFT, besides slightly overestimated HOMO and LUMO levels by DFT (Supplementary Table S1 and Supplementary Figure S20). Subsequently, the electrochemical properties of P1**–**P5 on a foam-nickel electrode were further examined and investigated through CV at different scan rates, galvanostatic charge–discharge (GCD) curves, and electrochemical impedance spectroscopy (EIS) and are summarized in Table 1 and Supplementary Figures S3–S7. The keeped shapes of current density–potential profiles at gradually increasing scan rates from 5, 10, 20, 50, 100, to 200 mV s^−1^ demonstrated their fast redox behaviors and high rate charge/discharge performances (Yigit and Gullu, 2018). For these polymers, their oxidation peak current densities were nearly linear with the corresponding scan rates (Supplementary Figure S8), suggesting that all polymers immobilized on the electrode surfaces very well and the redox processes are non-diffusional-controlled (Li et al., 2016b). From the shapes of CV and GCD curves (Supplementary Figures S3–S7), it can be seen that all polymers **P1–P5** exhibited obvious pseudocapacitive energy storage properties. The measured specific capacitances of the polymers (**P1–P5**)/foam-nickel electrode were calculated to be 70, 68, 75, 51, and 25 F/g (at 100 mV s^−1^) with 19, 10, 21, 26, and 69% of capacity retention after 1,000 cycles, respectively. These specific capacitances were higher than those of some other conducting polymer electrodes deposited by solution processing methods (Sun et al., 2020a; Sun et al., 2020b), probably owing to the excellent redox activity of EDOT. Together with the analysis of molecular structures, these data illustrate that the introduction of electron-donating units into the polymer backbone can improve the charge storage capacity, and the different cycle stabilities might be due to their different structure properties and/or microstructures. The GCD curves of **P1–P5** at various constant current densities exhibited deviation from the triangular geometry with a linear charge/discharge process and slight distortion, suggesting the existence of electrochemical double-layer behaviors, besides pseudocapacitive contribution by CPs. In contrast, the IR_drop_ values of **P3–P5** were much smaller than those of **P1–P2**, implying a small internal resistance which was probably attributed to their less twisted conformation. The Nyquist plots of all polymers exhibited a straight line in the low-frequency range with a negligible semicircle in the high-frequency range (Supplementary Figure S9), indicating the fast charge transfer between the electrode and the electrolyte surface (Zhang et al., 2016).

It is known that the electrochemical properties are closely related to the surface features and internal stacking characteristics (Fong et al., 2017). Thus, AFM and XRD characterization were performed to measure the surface morphologies and molecular packing patterns of these redox polymers, respectively. As demonstrated by AFM and shown in Supplementary Figures S10–S12, all the films of P1–P5 exhibited continuous and uniform morphologies, indicating homogeneous films obtained by solution processing methods, which is beneficial to the improvement of charge transfer. From the distinct XRD patterns of P5 (Supplementary Figure S13), diffraction peaks were observed at 2*θ* = 3.43, 6.79, 9.94, and 25.54°, corresponding to *d*-spacings of 25.73, 13.01, 8.89, and 3.48 Å, respectively, representing the existence of lamellar stacking and π–π stacking of polymer main chains. With the lowest roughness and best XRD crystallinity of **P5** among all polymers, the relatively good cyclic stability could be attributed to the homogeneous surface and well-ordered stacking of molecules in films. All the thermal degradation temperatures (*T*_d_) over 300°C of **P1–P5** at 5% weight loss manifested their good thermal stability (Supplementary Figure S14), which is conducive to further annealing processing and device applications.

To further investigate the influence of molecular structures on their cyclic stability, geometry optimizations of two repeating units at neutral, 50% doped, and 100% doped states were carried out by DFT calculations (see the Supplementary Material for details) (Murto et al., 2020). The geometry changes between neutral, 50% doped, and 100% doped states are measured by the root-mean-squared displacement (RMSD), and the maximum RMSD values are summarized in Table 1. Obviously, the minimum RMSD (0.1129) of **P5** implied the least backbone distortions at different oxidative states, which is favorable to electrochemical stability during the charge/discharge cycles and is responsible for the good cycling stability of **P5** compared to those of the others. It is interesting that the thiophene backbone of **P3** featured coplanar conformations besides obvious rotations of methoxy groups at neutral, 50% doped, and 100% doped states, thus causing a large RMSD of 0.5437. The increased RMSD means large volumetric changes induced by swelling and shrinkage during the charge/discharge process, which might be the reason for the greatly improved cycling stability when the alkoxy groups attached to thiophene were fixed by forming a seven-membered ring (Osterholm et al., 2016).

## Conclusion

In this work, a series of novel soluble EDOT-based conjugated copolymers consisting of electron-rich or electron-deficient units were synthesized steadily in moderate to excellent yields (60–98%) with medium to high molecular weights (*M*_n_ = 3,100–94,000 Da) through direct (hetero)arylation polymerization. Owing to the structural diversity of obtained EDOT-based copolymers, the varied absorption spectra, specific capacitance, and capacity retention were observed, which should be attributed to the electronic characteristics of various units introduced, molecular torsions in the polymer backbone, and thus resultant different surface morphologies and molecular packings. Clear redshifts in absorption of **P3**, **P4**, and **P5** were observed, due to their good coplanarity and/or ICT property. When using a foam-nickel electrode drop-coated with the polymer as the working electrode in a three-electrode cell, good specific capacitances of 70, 68, 75, 51, and 25 F/g (at 100 mV s^−1^) with 19, 10, 21, 26, and 69% of capacity retention after 1,000 cycles for **P1–P5** were obtained, respectively, owing to the good redox activity of the EDOT unit. The results of our present study confirm that appropriate electronic characteristics, fast charge transfer, good coplanarity, low RMSD between different oxidative states, homogeneous surface, and well-ordered molecular stacking could improve the cycling stability of polymeric capacitive performance which is still one of the main obstacles in further applications of supercapacitors based on π-conjugated polymers. It is anticipated that such study will provide a novel strategy for the molecular design of π-conjugated polymers regarding electrochemical properties.

## Data Availability

The original contributions presented in the study are included in the article/Supplementary Material, and further inquiries can be directed to the corresponding author.
