# Supplementary material for: Physical and Electrochemical Properties of Soluble 3,4-Ethylenedioxythiophene (EDOT)-Based Copolymers Synthesized via Direct (Hetero)Arylation Polymerization
Source: Front Chem. 2021 Oct 29;9:753840. doi: 10.3389/fchem.2021.753840 (PMC8586465; doi:10.3389/fchem.2021.753840)
Supplement: Supplementary file 1 [file DataSheet1.pdf]

Supplementary Material

Physical and Electrochemical Properties of Soluble 3,4-Ethylenedioxythiophene (EDOT)-Based Copolymers Synthesized via Direct Hetero(Arylation) Polymerization

Qiang Guo[1], Jincheng Zhang[1], Xiaoyu Li[2], Heqi Gong[1], Shuanghong Wu[1] and Jie Li[1*]

[1]College of Optoelectronic Engineering, Chengdu University of Information Technology, Chengdu, China

[2]Southwest University of Science and Technology, Mianyang, China

[3]School of Optoelectronic Science and Engineering, University of Electronic Science and Technology of China, Chengdu, China

*** Correspondence:**
Jie Li
lijie@cuit.edu.cn

Table of Contents

I. General remarks...S2

II. Synthesis procedures...S3

III. Photophysical, electrochemical, AFM, XRD and TGA Properties...............................S6

IV. Theoretical calculations..S12

V. References..S20

VI. Copies of ^{1}H NMR, MALTI-TOF Mass spectra and GPC elution curves of **P1-P5**...... S21

I. General remarks

1. Materials

Unless otherwise noted, all reagents and extra dry solvents *N*,*N*-dimethylacetamide (DMAc, stored with molecular sieves) were obtained from commercial suppliers and used without further purification. Unless otherwise indicated, all syntheses and manipulations were carried out under N_2 atmosphere. 2,7-Dibromo-9,9-didecyl-9H-fluorene[S1], 3,6-dibromo-*N*-(2-ethylhexyl)carbazole[S2], 3,4-bis(2-ethylhexyloxy)thiophene[S3], 4,7-dibromo-2-(2-ethylhexyl)benzotriazole[S4] and 1,3-dibromo-5-(2-ethylhexyl)thieno[3,4-*c*]pyrrole-4,6-dione[S5] were prepared according to the literature.

2. Instrumentation

NMR spectra were obtained on a Varian Inova 400 spectrometer. The ^{1}H NMR (400 MHz) chemical shifts were measured relative to $CDCl_3$ as the internal reference ($CDCl_3$: δ = 7.26 ppm). Gel permeation chromatography (GPC) measurements were performed on a HLC-8320GPC system using THF as eluent and polystyrene as standards at a column temperature of 40 °C. MALDI-TOF-MASS spectra were measured with Shimadzu Biotech Axima Performance MALDI TOF Mass Spectrometer. UV-vis-NIR spectra were recorded on Shimadzu UV-2550 spectrophotometer. Cyclic voltammetry (CV) and galvanostatic charge-discharge (GCD) measurements were performed on CS350H in a three-electrode electrochemical cell, using an Ag/Ag^+ (0.01 M of $AgNO_3$ in acetonitrile) reference electrode, a platinum wire counter electrode, and a Pt or foam-nickel electrode drop-coated with polymer as working electrode. The working electrode was coated with the polymer film (0.1 mg) by drop-casting a polymer solution in chloroform. The electrochemical impedance spectroscopy (EIS) were recorded in the frequency range from 100 kHz to 0.1 Hz at the open circuit potential by applying a small potential of 5 mV sinusoidal signal. Morphological images of polymer thin films deposited by spray coating were acquired by a Being Nano-Instruments BY3000 atomic force microscope (AFM). XRD experiments were performed on a Bruker D8 Advance X-ray diffractometer with Cu Kα radiation at a generator voltage of 40 kV and a current of 30 mA. Thermogravimetric analysis (TGA) was carried out using PerkinElmer STA 8000 at a heating rate of 10 °C·min^{-1} under N_2 atmosphere.

II. Synthesis procedures

General Direct coupling polycondensation procedure

A flame-dried Schlenk tube with a magnetic stir bar was charged with mixture of $Pd(OAc)_2$ (4.5 mg, 0.02 mmol), 1-adamantanecarboxylic acid(1-AdCOOH, 36.0 mg, 0.2 mmol), K_2CO_3 (138 mg, 1.0 mmol), dibromo-aromatic compound (0.40 mmol), 3,4-ethylenedioxythiophene (EDOT) (56.9 mg, 0.40 mmol), anhydrous DMAc (2.0 mL) under an N_2 atmosphere. The resulting mixture was stirred for several minutes at room temperature, and then heated at 100 °C for 24 h. After cooling to room temperature, the reaction mixture was poured into a mixture of water and methanol. The resulting solid was filtered and subjected to Soxhlet extraction in methanol, acetone and hexane, respectively, for the removal of low molecular weight materials and impurities. The remaining polymer was extracted with chloroform, precipitated again from methanol, filtered, washed with methanol and dried under vacuum.

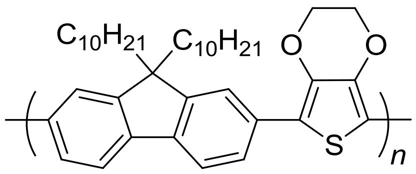

Poly[(9,9-didecylfluorene-2,7-diyl)-(3,4-ethylenedioxythiophene-2,5-diyl)] (P1)

According to the general procedure, **P1** was obtained as a yellow solid (230 mg, 98% yield). M_n = 94000, PDI = 1.85. ^1H NMR (400 MHz, $CDCl_3$): δ = 7.82 (d, J = 8.0 Hz, 2H), 7.68-7.71 (m, 4H), 4.45 (s, 4H), 2.05 (br, 4H), 1.10-1.25 (m, 32 H), 0.82 (t, J = 6.8 Hz, 6H) ppm. A set of peaks at intervals of the molecular weight of a repeating unit (Mass: 584) can be found in the MALDI-TOF mass spectra.

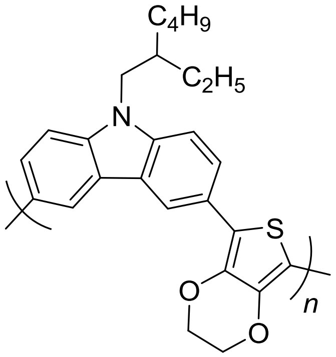

Poly[(*N*-(2-ethylhexyl)carbazole-3,6-diyl)-(3,4-ethylenedioxythiophene-2,5-diyl)] (P2)

According to the general procedure, **P2** was obtained as an olive green solid (100 mg, 60% yield). M_n = 3100, PDI = 1.32. ^{1}H NMR (400 MHz, $CDCl_3$): δ = 8.46 (br, 2H), 7.91 (br, 2H), 7.41 (br, 2H), 4.41 (br, 4H), 3.98 (br, 2H), 1.98 (br, 1H), 1.26 (br, 8H), 0.86-0.96 (m, 6H) ppm. A set of peaks at intervals of the molecular weight of a repeating unit (Mass: 417) can be found in the MALDI-TOF mass spectra.

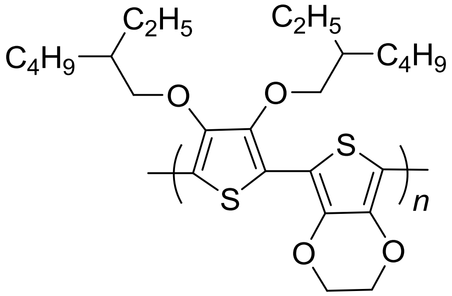

Poly[(3,4-bis(2-ethylhexyl)thiophene-2,5-diyl)-(3,4-ethylenedioxythiophene-2,5-diyl)] (P3)

According to the general procedure, **P3** was obtained as a black solid (144 mg, 75% yield). M_n = 26900, PDI = 1.39. ^{1}H NMR (400 MHz, $CDCl_3$): δ = 4.36 (br, 4H), 3.96 (br, 4H), 1.88-2.00 (m, 2H), 1.30-1.51 (m, 16H), 0.86-1.00 (m, 12H) ppm. A set of peaks at intervals of the molecular weight of a repeating unit (Mass: 478) can be found in the MALDI-TOF mass spectra.

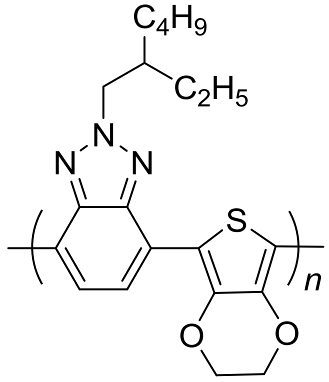

Poly[(2-(2-ethylhexyl)benzotriazole-4,7-diyl)-(3,4-ethylenedioxythiophene-2,5-diyl)] (P4)

According to the general procedure, **P4** was obtained as a black solid (99 mg, 67% yield). M_n = 7000, PDI = 1.74. ^{1}H NMR (400 MHz, $CDCl_3$): δ = 8.21 (br, 2H), 4.82 (br, 2H), 4.49 (br, 4H), 2.42 (br, 1H), 1.26-1.42 (m, 8H), 0.82-0.88 (m, 6H) ppm. A set of peaks at intervals of the molecular weight of a repeating unit (Mass: 369) can be found in the MALDI-TOF mass spectra.

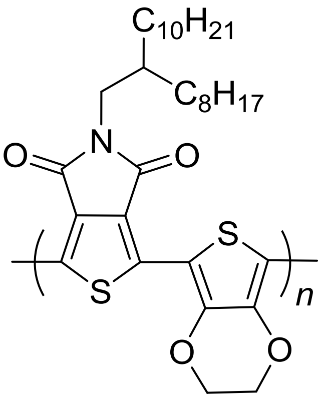

Poly[(5-(2-octyldodecyl)-5*H*-thieno[3,4-*c*]pyrrole-4,6-dione-1,3-diyl)-(3,4-ethylenedioxythiophene-2,5-diyl)] (P5)

According to the general procedure, **P5** was obtained as a black solid (197 mg, 86% yield). M_n = 11000, PDI = 1.65. ^{1}H NMR (400 MHz, $CDCl_3$): δ = 4.34 (br, 4H), 3.64 (br, 2H), 2.03 (br, 1H), 1.25 (br, 32 H), 0.87 (br, 6H) ppm. A set of peaks at intervals of the molecular weight of a repeating unit (Mass: 571) can be found in the MALDI-TOF mass spectra.

III. **Photophysical, electrochemical, AFM XRD and TGA Properties**

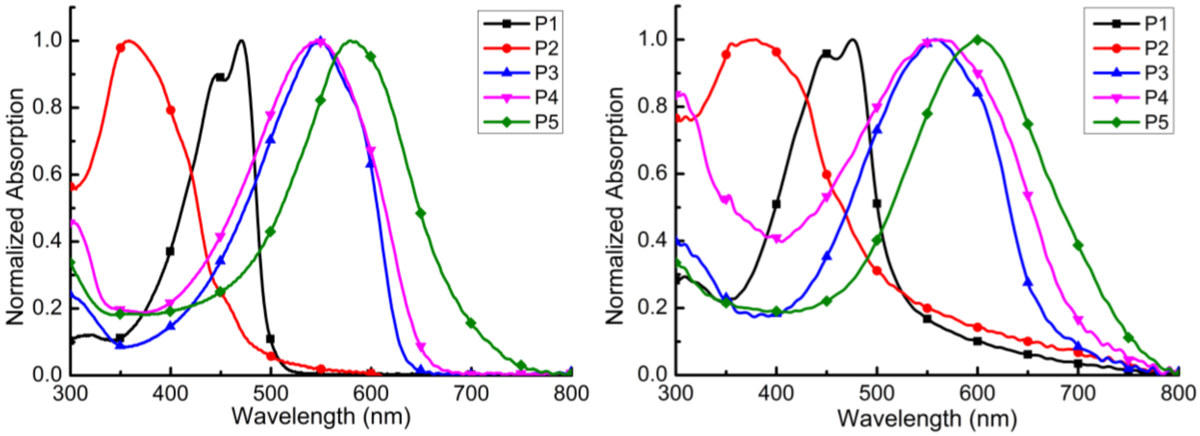

s

Figure S1 The UV-vis spectra of **P1-P5** in diluted CH_2Cl_2 solution (left) and in thin films deposited by spray coating on quartz plates (right).

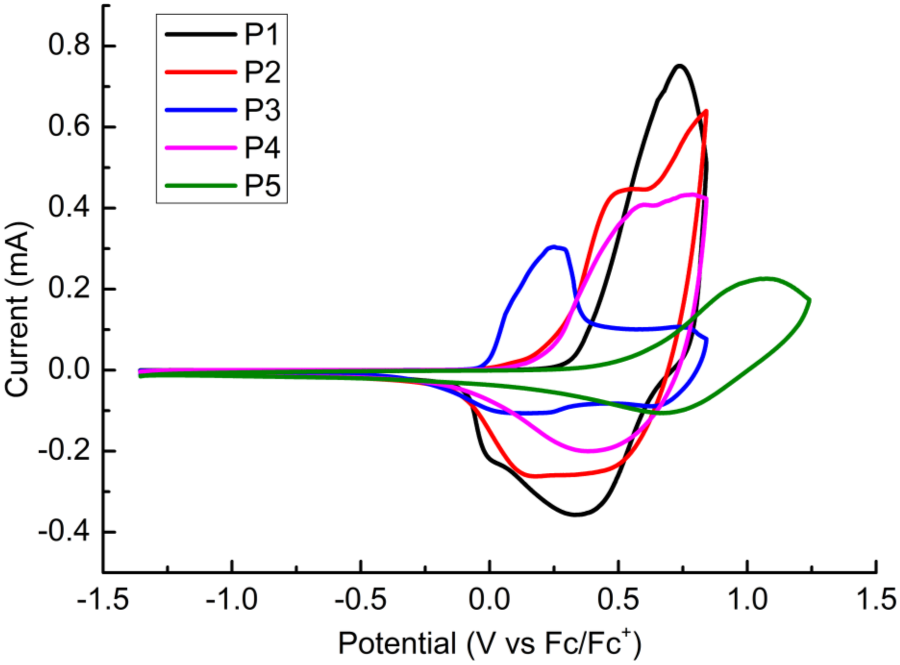

Figure S2 CV curves of **P1-P5** at a large range and a scan rate of 100 $mV{\cdot}s^{-1}$

Table S1 Estimated HOMO, LUMO energy levels by electrochemical measurments and DFT calculations

Polymer	Experimetal data				Calculated data[e)]	
	$E_{ox,onset}$ [V][a)]	E_g [eV][b)]	HOMO [eV][c)]	LUMO [eV][d)]	HOMO [eV]	LUMO [eV]
**P1**	0.37	2.38	−5.17	−2.79	−4.55	−1.57
**P2**	0.26	2.48	−5.06	−2.58	−4.36	−1.05
**P3**	−0.01	1.86	−4.79	−2.93	−3.86	−1.65
**P4**	0.22	1.76	−5.02	−3.26	−4.33	−1.79
**P5**	0.55	1.65	−5.35	−3.70	−4.75	−2.46

a) Using Pt disk electrode as the working electrode and $E_{ox,onset}$ were estimated from the onset of the oxidation peak with respect to a standard ferrocene/ferrocenium (Fc/Fc^{+}) redox couple. b) Estimated from the absorption band edge in film state, $E_g = 1240/\lambda_{onset}$ (eV). c) HOMO = − (4.80 + $E_{ox,onset}$) (eV). d) LUMO = HOMO + E_g (eV). e) Calculated by using DTF theory (B3LYP function) at 6-31G* level (*vide infra*).

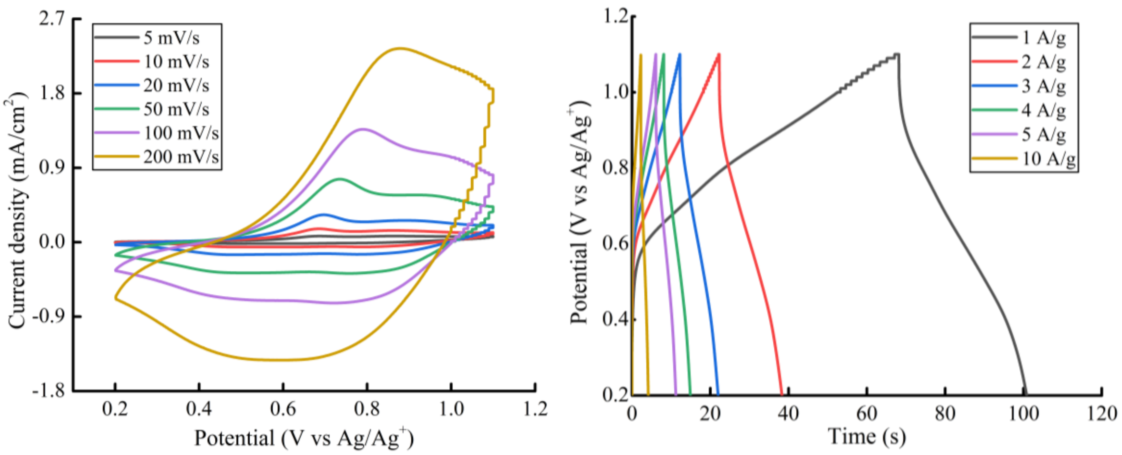

Figure S3 CV curves of **P1** at different scan rates (left) and GCD curves of **P1** at different current densities (right)

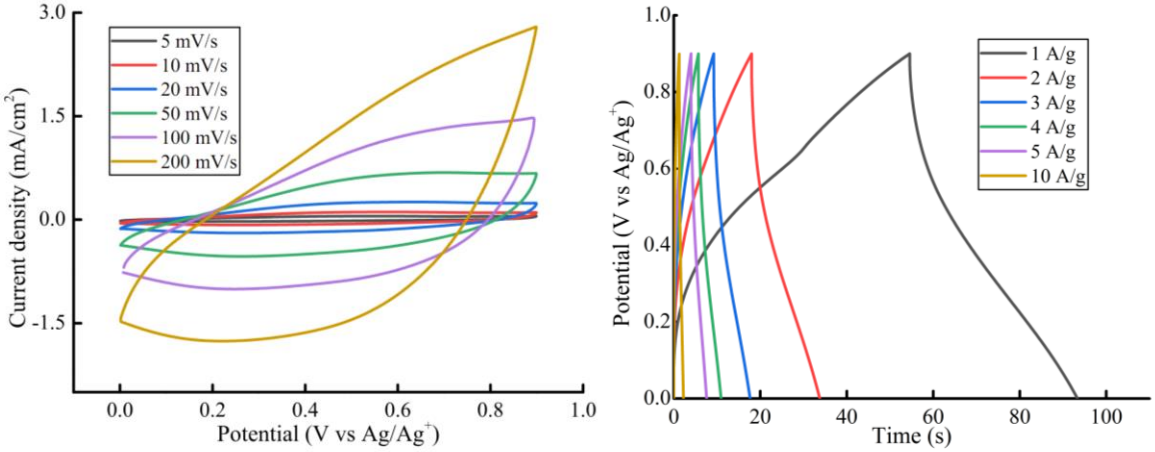

Figure S4 CV curves of **P2** at different scan rates (left) and GCD curves of **P2** at different current densities (right)

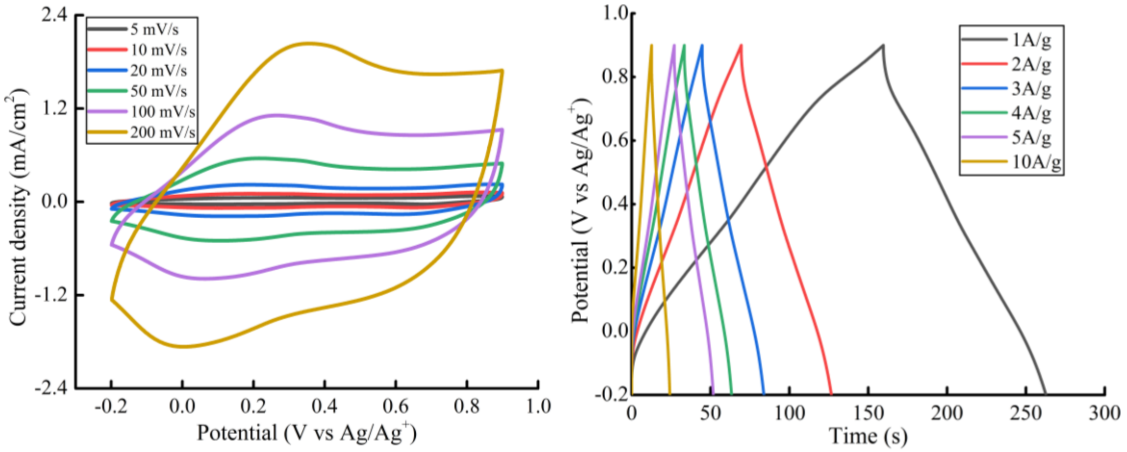

Figure S5 CV curves of **P3** at different scan rates (left) and GCD curves of **P3** at different current densities (right)

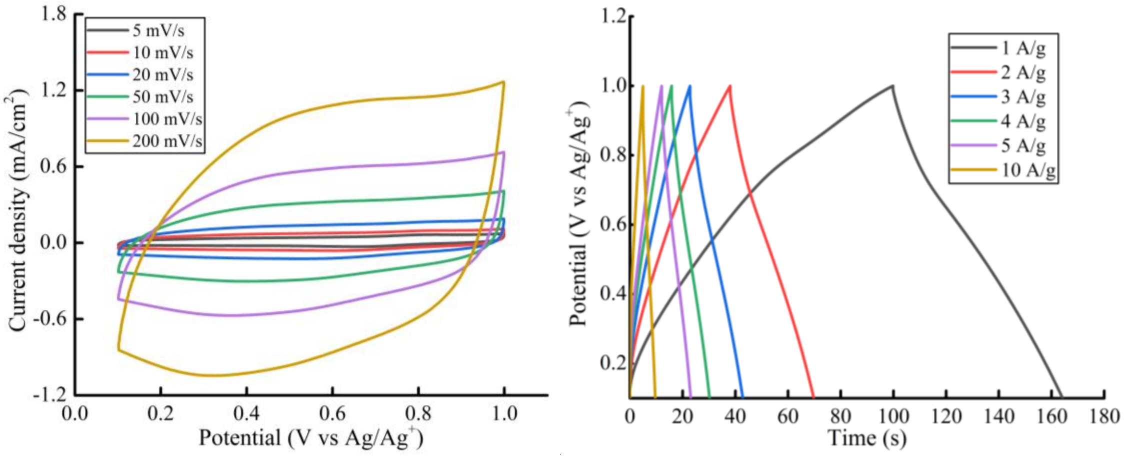

Figure S6 CV curves of **P4** at different scan rates (left) and GCD curves of **P4** at different current densities (right)

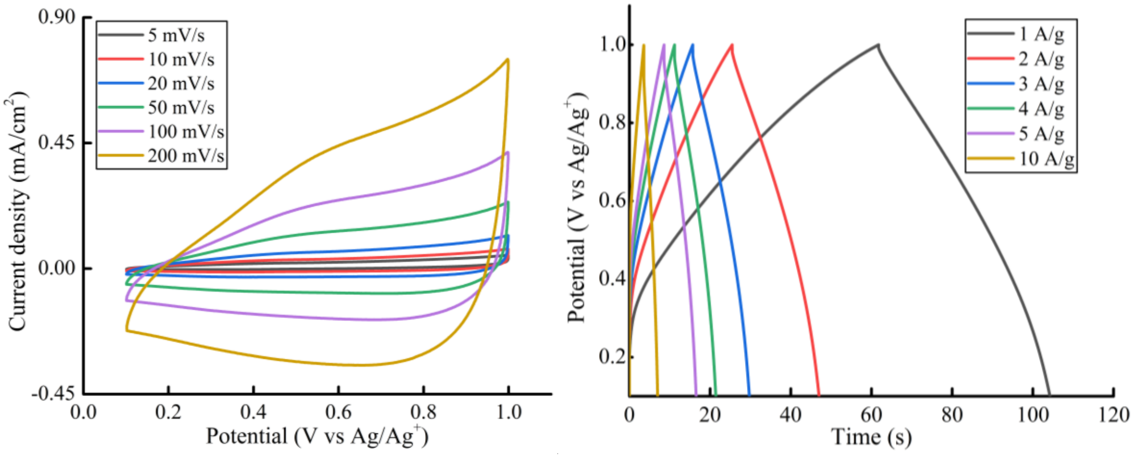

Figure S7 CV curves of **P5** at different scan rates (left) and GCD curves of **P5** at different current densities (right)

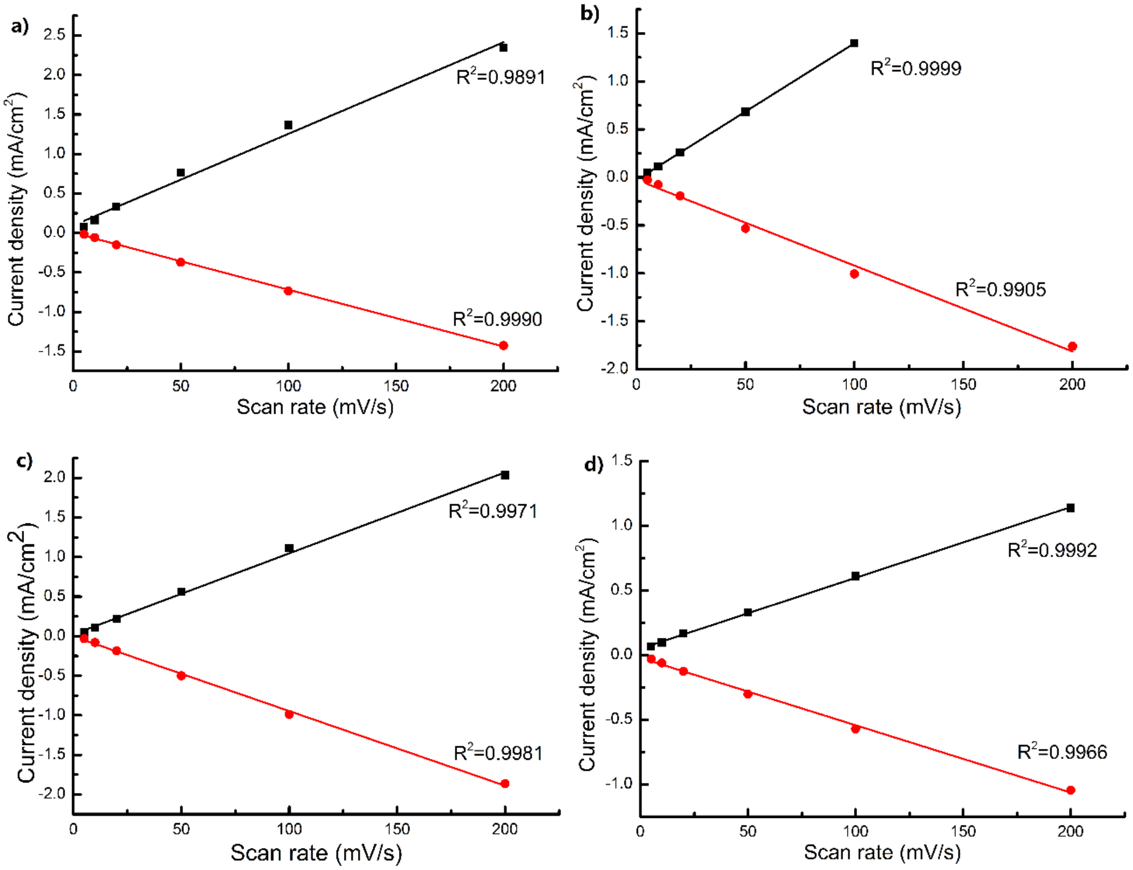

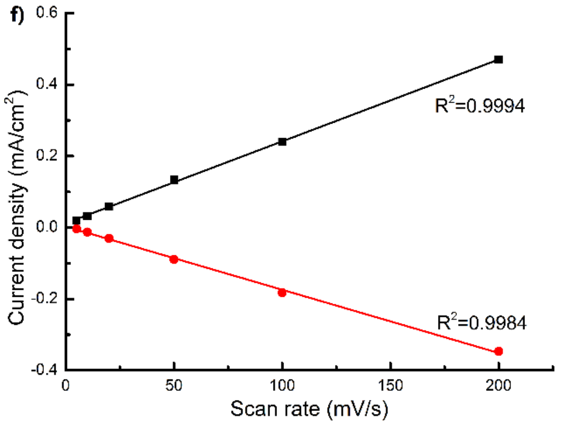

Figure S8 Linear relationship between peak current densities and scan rate of a) **P1**, b) **P2**, c) **P3**, d) **P4** and e) **P5**.

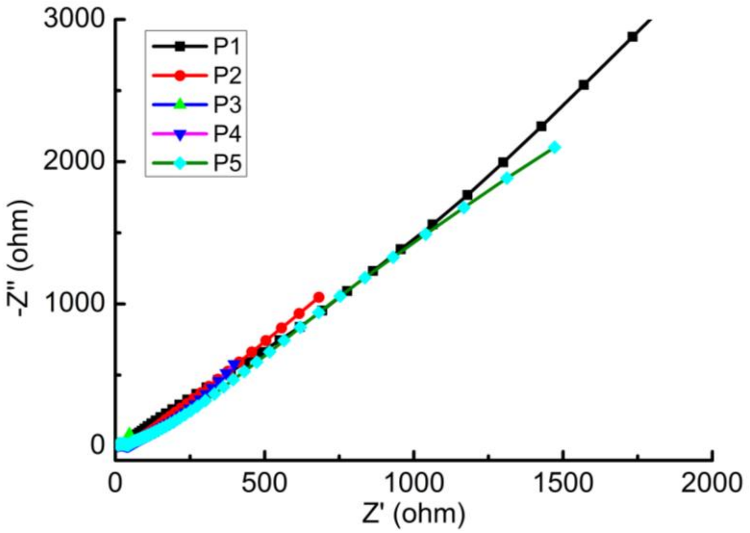

Figure S9 Nyquist plots of **P1-P5** polymer films on foam-nickel electrode

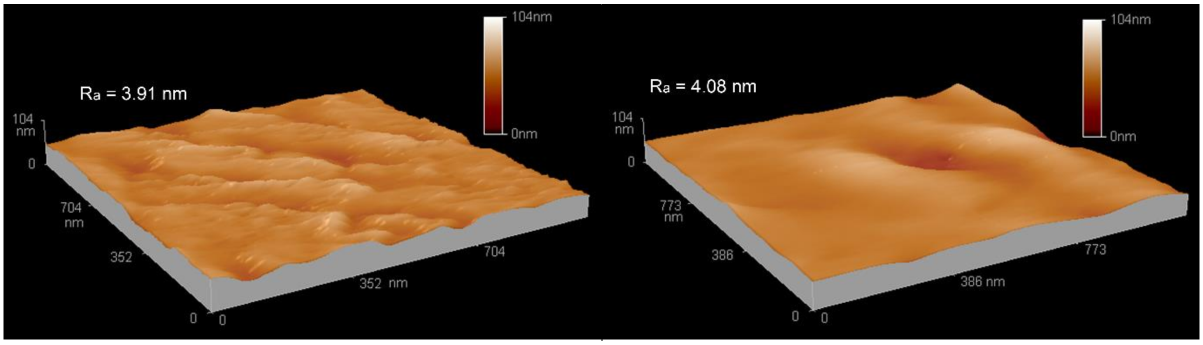

Figure S10 3D AFM images of **P1** (left) and **P2** (right) thin films deposited by spray coating. (Ra means "surface average roughness")

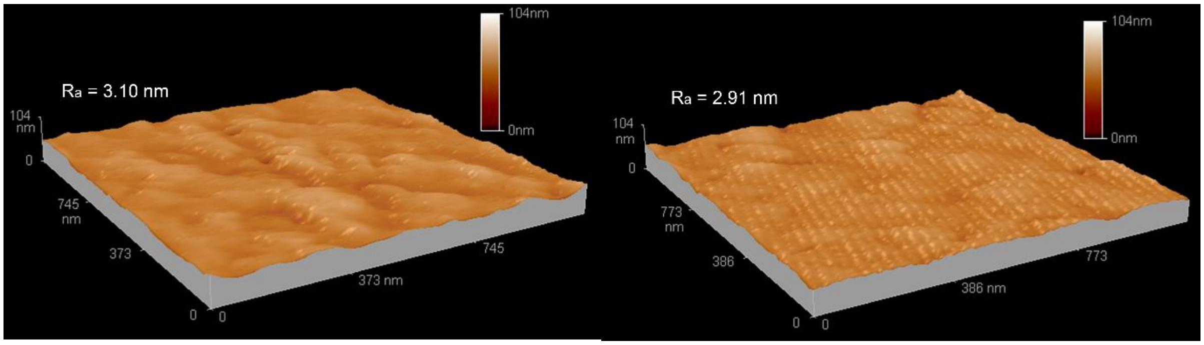

Figure S11 3D AFM images of **P3** (left) and **P4** (right) thin films deposited by spray coating. (Ra means "surface average roughness")

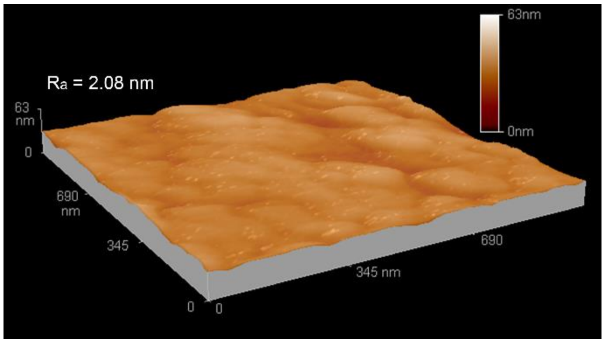

Figure S12 3D AFM images of **P5** thin films deposited by spray coating. (Ra means "surface average roughness")

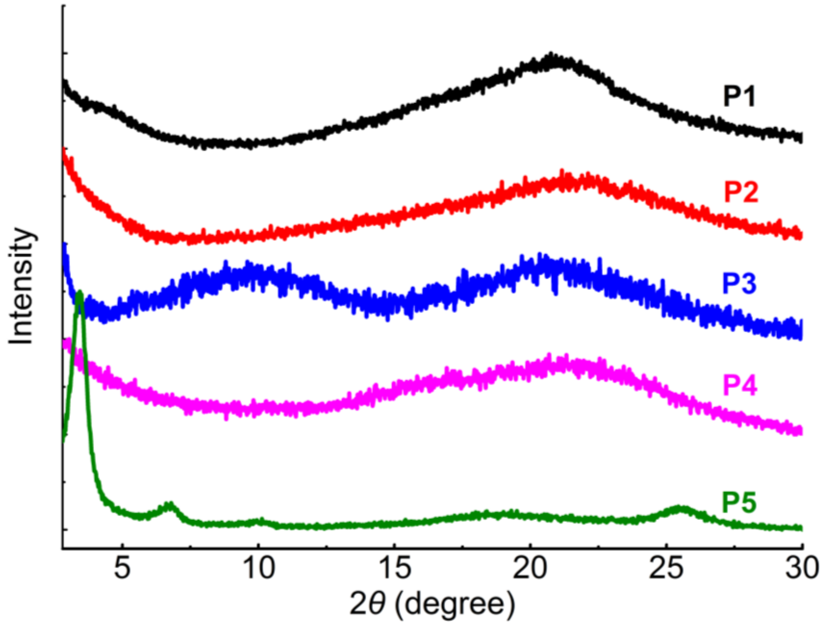

Figure S13 XRD patterns of **P1-P5** deposited by drop coating on glass slide.

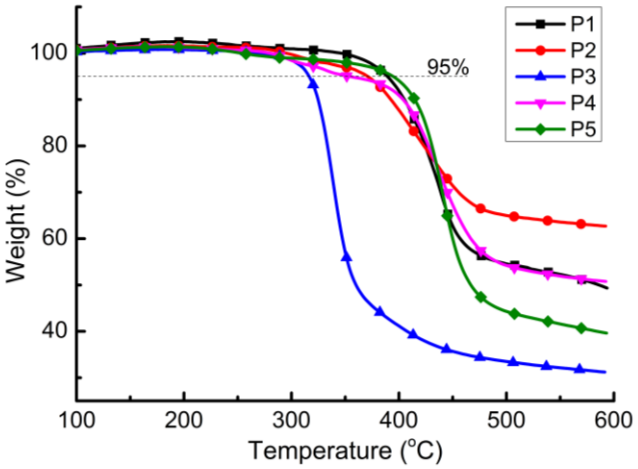

Figure S14 TGA curves of the polymers **P1-P5**.

IV. Theoretical calculations

The theoretical calculations were carried out by using Gaussian 16 A.03 package[S6]. Ground state geometries of structures were fully optimized by using ωB97XD hybrid functional of density functional theory (DFT) with 6-31G(d) basis set.[S7] Molecular models for polymers **P1-P5** were shorten as dimers within two repeating units to balance the effectiveness in predicting geometries of conjugated polymers and work load practical for computation. Alkyl side chains were replaced by methyl groups due to its limited contribution to the electronic structures of skeletons. To predict geometries of doped dimers, one and two electron oxidations correspond to 50 and 100% doping, respectively.[S8] Corresponding to the 50% doped and 100% doped states, the calculation parameters of charges and spin multiplicities were set as 1, 2 and 2, 3, respectively. The geometry changes between neutral, 50% doped and 100% doped states are measured by root-mean squared displacement (RMSD) with the expression of $\text{RMSD}=\sqrt{\frac{1}{N}\sum^{natom}\left[(x_i-x_i^{'})^2+(y_i-y_i^{'})^2+(z_i-z_i^{'})^2\right]}$. RMSD_{max} means the maximum RMSD value of geometry changes between neutral, 50% doped and 100% doped states, calculated by VMD program[S9]. The HOMO and LUMO energy levels of **P1-P5** were calculated by extrapolating the linear curve of orbital energy levels of oligomers against the empirical parameter $1/n^{1.5}$ (n was the number of monomer units). The orbital energy levels of oligomers were calculated by DFT at B3LYP/6-31G* level after fully optimized at ωB97XD/6-31G* level.

<table>
<tr><td rowspan="3">Geometry of dimer at neutral ground state</td><td>Face view</td><td>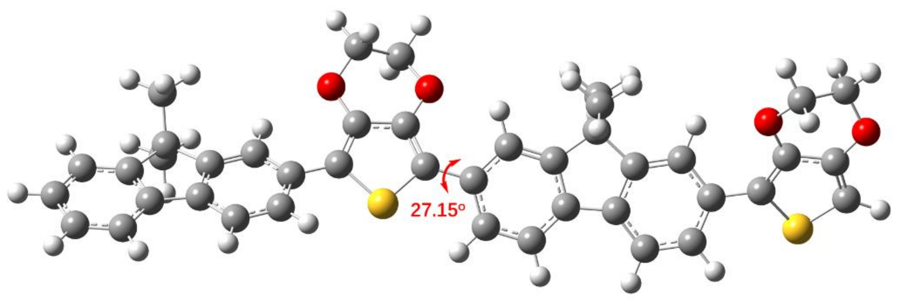
</td></tr>
<tr><td>Side View</td><td>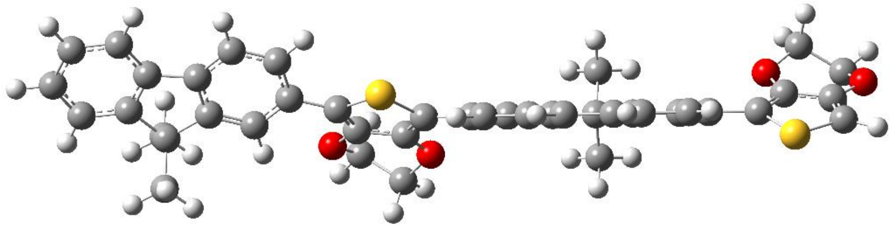</td></tr>
<tr><td>HOMO orbital distributions</td><td>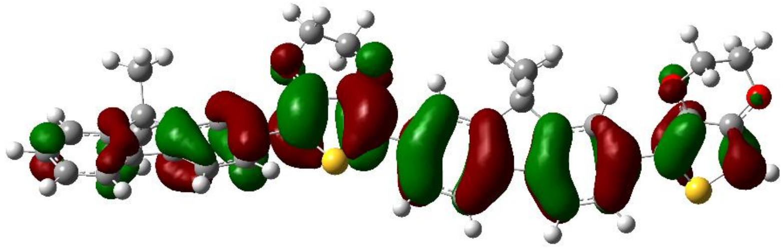</td></tr>
<tr><td rowspan="2">Overlap geometries of dimers at neutral, 50% and 100% doped state</td><td>Face view</td><td>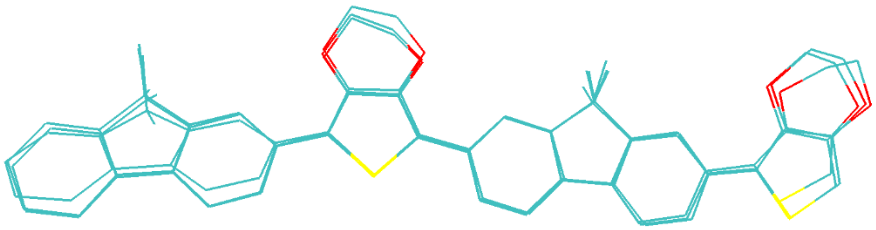</td></tr>
<tr><td>Side View</td><td>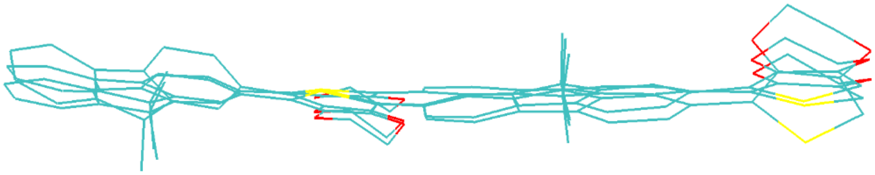</td></tr>
</table>

Figure S15 Geometry of dimer of **P1** at neutral ground state and overlap geometries of dimers at neutral, 50% and 100% doped state. To observe the changes of geometries between different states clearly, the hydrogens were hidden. (Maximum RMSD = 0.6324).

<table>
<tr><td rowspan="3">Geometry of dimer at neutral ground state</td><td>Face view</td><td>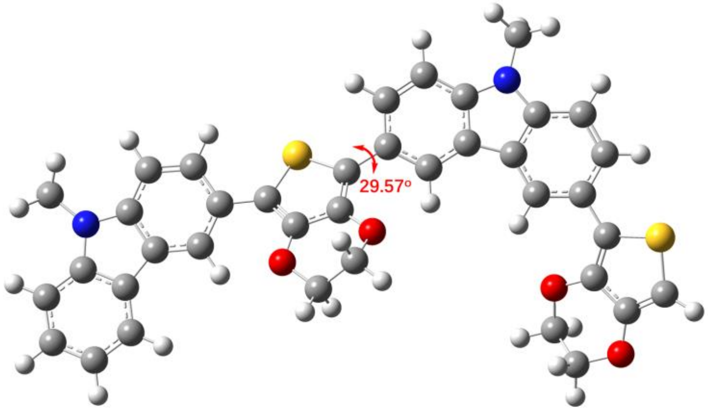</td></tr>
<tr><td>Side View</td><td>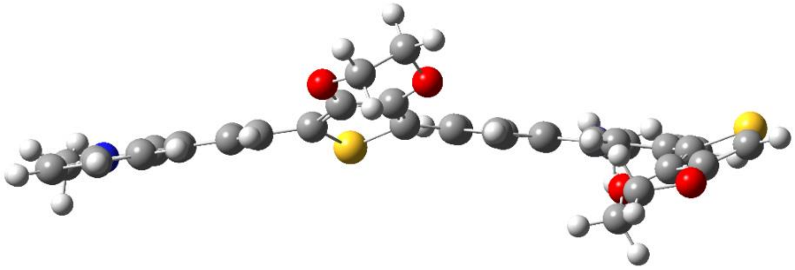</td></tr>
<tr><td>HOMO orbital distributions</td><td>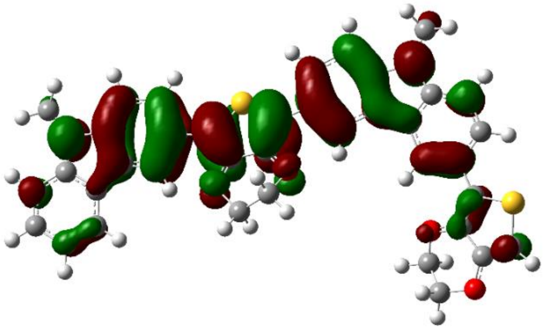</td></tr>
<tr><td>Overlap geometries of dimers at neutral, 50% and 100% doped state</td><td>Face view</td><td>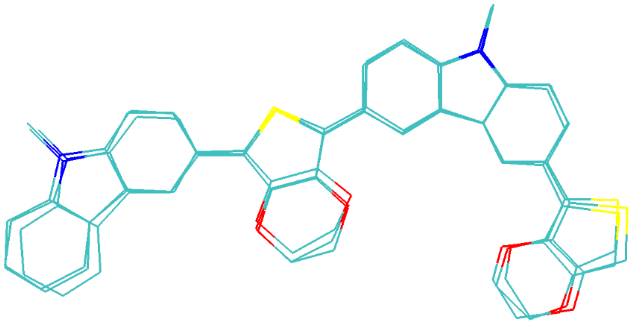</td></tr>
</table>

	Side View	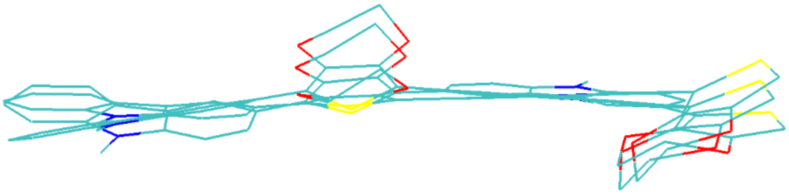

Figure S16 Geometry of dimer of **P2** at neutral ground state and overlap geometries of dimers at neutral, 50% and 100% doped state. To observe the changes of geometries between different states clearly, the hydrogens were hidden. (Maximum RMSD = 0.6485)

<table>
<tr><td rowspan="3">Geometry of dimer at neutral ground state</td><td>Face view</td><td>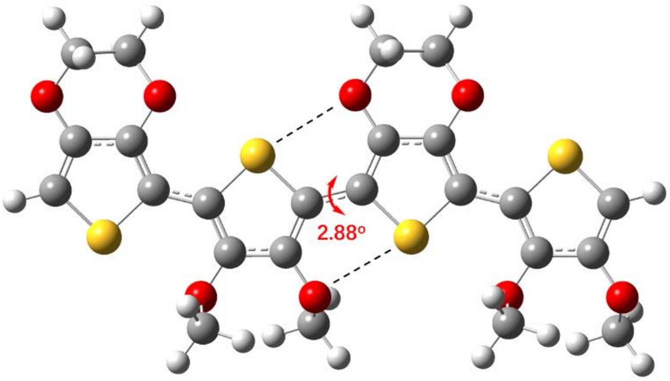</td></tr>
<tr><td>Side View</td><td>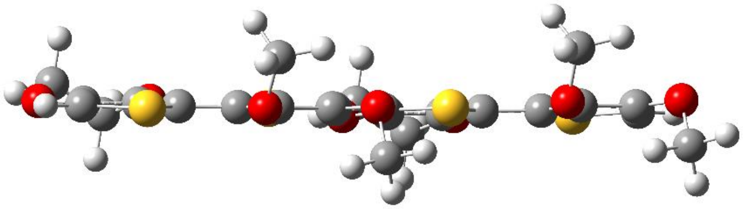</td></tr>
<tr><td>HOMO orbital distributions</td><td>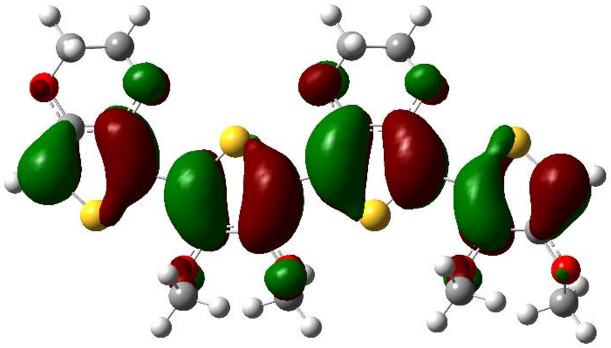</td></tr>
</table>

<table>
<tr><td rowspan="2">Overlap geometries of dimers at neutral, 50% and 100% doped state</td><td>Face view</td><td>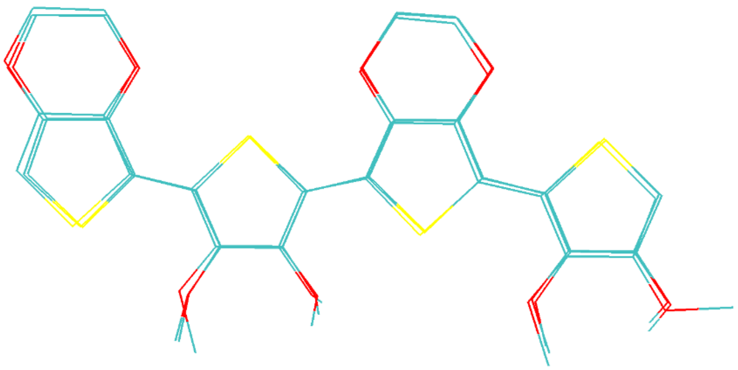</td></tr>
<tr><td>Side View</td><td>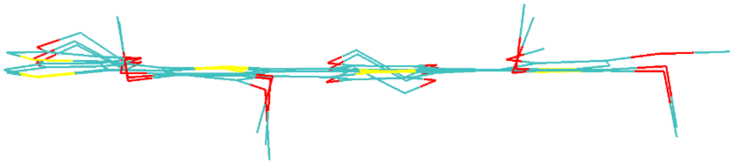</td></tr>
</table>

Figure S17 Geometry of dimer of **P3** at neutral ground state and overlap geometries of dimers at neutral, 50% and 100% doped state. To observe the changes of geometries between different states clearly, the hydrogens were hidden. (Maximum RMSD = 0.5437)

<table>
<tr><td>Geometry of dimer at neutral ground state</td><td>Face view</td><td>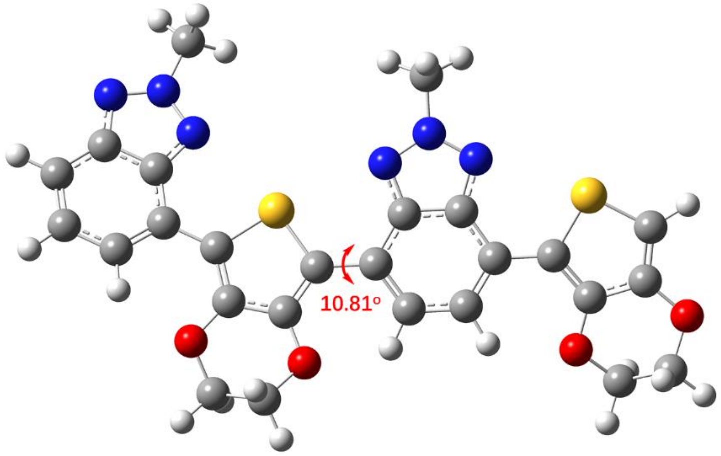
</td></tr>
</table>

	Side View	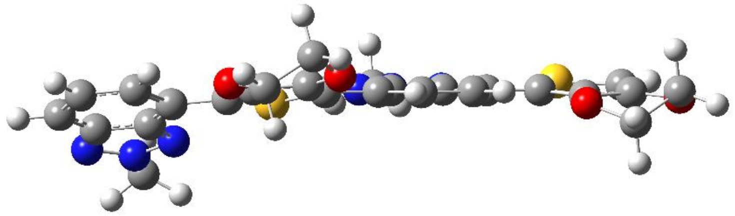
	HOMO orbital distributions	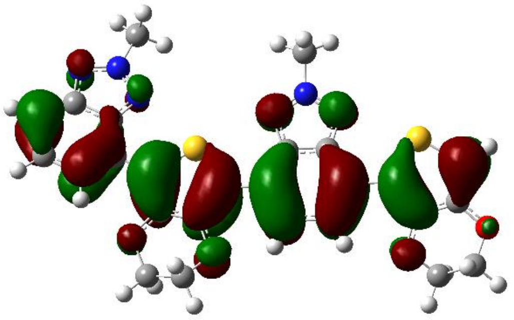
Overlap geometries of dimers at neutral, 50% and 100% doped state	Face view	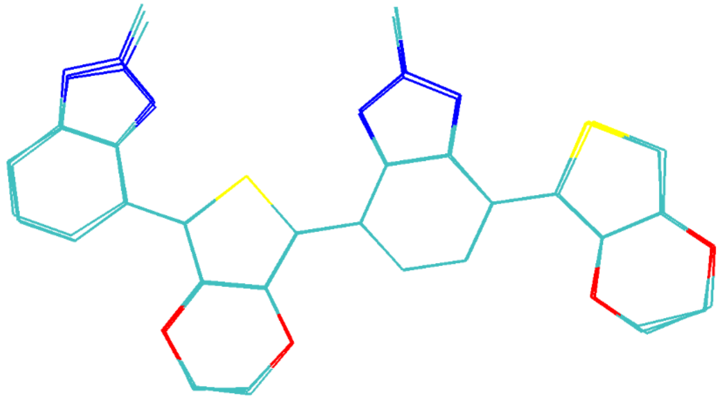
	Side View	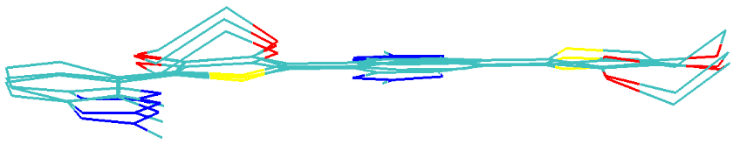

Figure S18 Geometry of dimer of **P4** at neutral ground state and overlap geometries of dimers at neutral, 50% and 100% doped state. To observe the changes of geometries between different states clearly, the hydrogens were hidden. (Maximum RMSD = 0.3827)

Geometry of dimer at neutral ground state	Face view	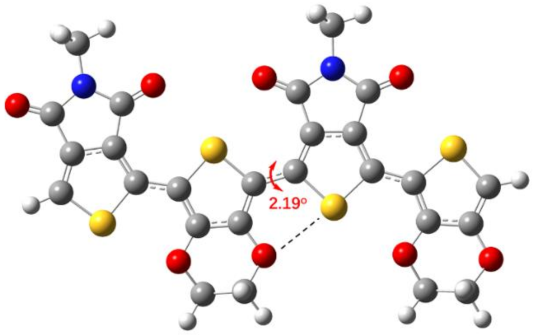
	Side View	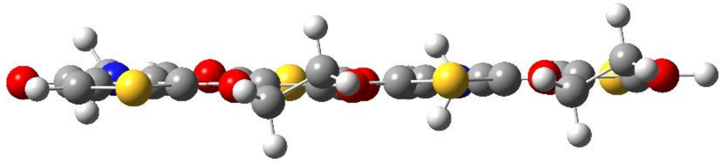
	HOMO orbital distributions	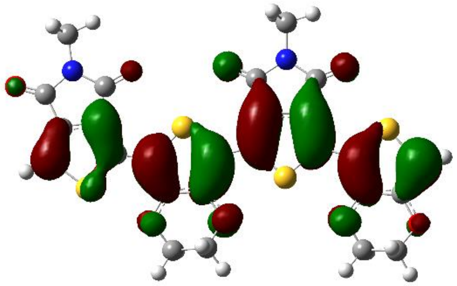
Overlap geometries of dimers at neutral, 50% and 100% doped state	Face view	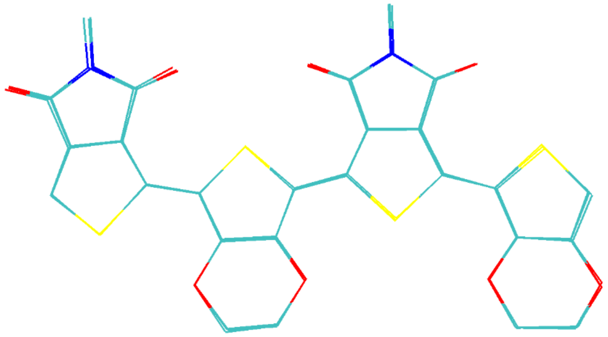
	Side View	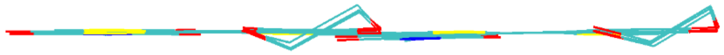

Figure S19 Geometry of dimer of **P5** at neutral ground state and overlap geometries of dimers at neutral, 50% and 100% doped state. To observe the changes of geometries between different states clearly, the hydrogens were hidden. (Maximum RMSD = 0.1129)

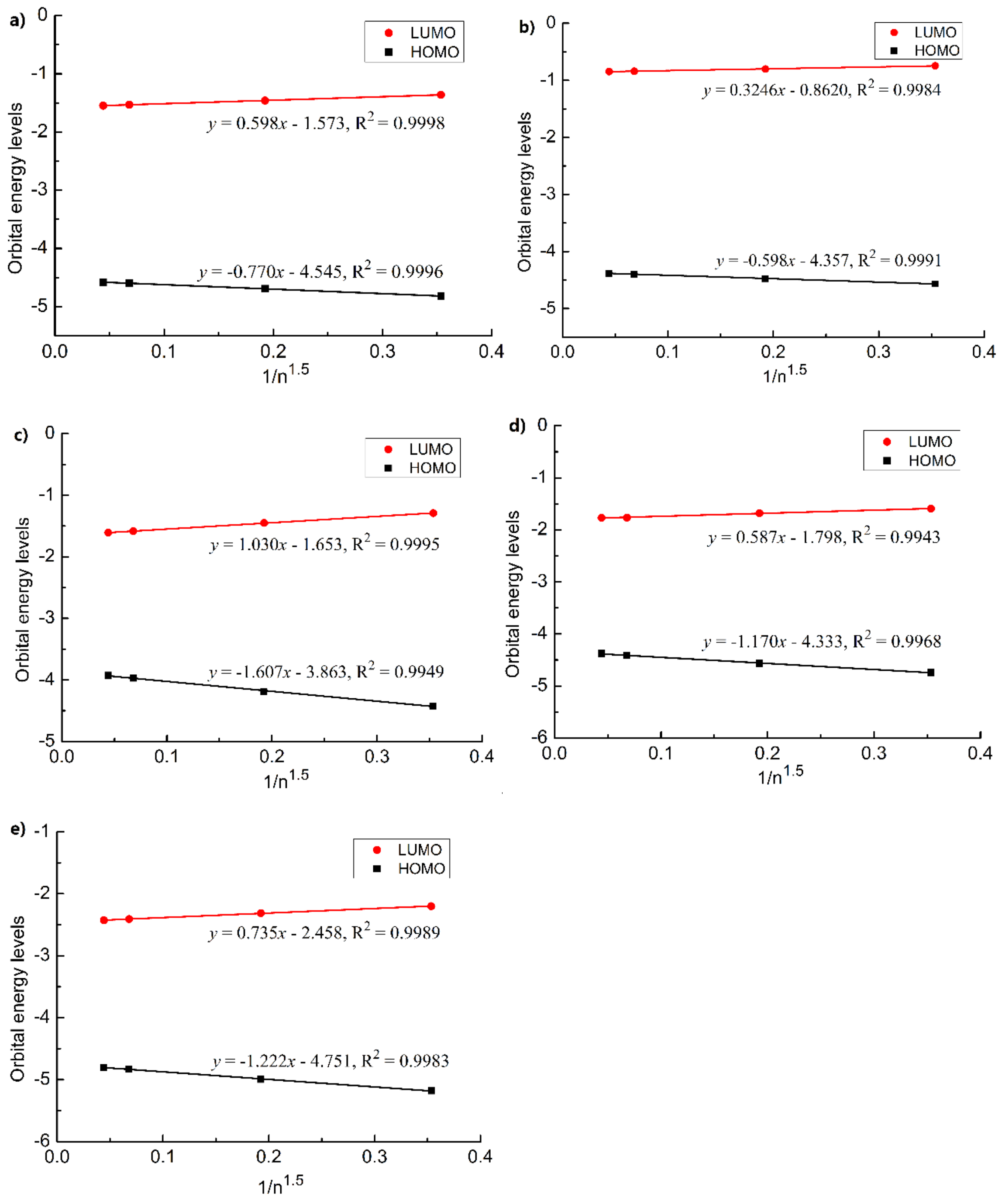

Figure S20 Calculated orbital energy levels (HOMO and LUMO) for a) **P1**, b) **P2**, c) **P3**, d) **P4** and e) **P5** by DFT, respectively. The lines correspond to linear fitting of the energy levels vs.$1/n^{1.5}$.

V. References

[S1] D. Bléger, A. Ciesielski, P. Samorì, S. Hecht, *Chem. Eur. J.* **2010**, *16*, 14256.

[S2] Y. Zhu, A. R. Rabindranath, T. Beyerlein, B. Tieke, *Macromolecules* **2007**, *40*, 6981.

[S3] W. T. Neo, K. H. Ong, T. T. Lin, S.-J. Chua, J. Xu, *J. Mater. Chem. C*, **2015**, *3*, 5589.

[S4] A. Tanimoto, T. Yamamoto, *Macromolecules* **2006**, *39*, 3546.

[S5] A. E. Labban, J. Warnan, C. Cabanetos, O. Ratel, C. Tassone, M. F. Toney, P. M. Beaujuge, *ACS Appl. Mater. Interfaces* **2014**, *6*, 19477.

[S6] Gaussian 16, Revision A.03, M. J. Frisch, G. W. Trucks, H. B. Schlegel, G. E. Scuseria, M. A. Robb, J. R. Cheeseman, G. Scalmani, V. Barone, G. A. Petersson, H. Nakatsuji, X. Li, M. Caricato, A. V. Marenich, J. Bloino, B. G. Janesko, R. Gomperts, B. Mennucci, H. P. Hratchian, J. V. Ortiz, A. F. Izmaylov, J. L. Sonnenberg, D. Williams-Young, F. Ding, F. Lipparini, F. Egidi, J. Goings, B. Peng, A. Petrone, T. Henderson, D. Ranasinghe, V. G. Zakrzewski, J. Gao, N. Rega, G. Zheng, W. Liang, M. Hada, M. Ehara, K. Toyota, R. Fukuda, J. Hasegawa, M. Ishida, T. Nakajima, Y. Honda, O. Kitao, H. Nakai, T. Vreven, K. Throssell, J. A. Montgomery, Jr., J. E. Peralta, F. Ogliaro, M. J. Bearpark, J. J. Heyd, E. N. Brothers, K. N. Kudin, V. N. Staroverov, T. A. Keith, R. Kobayashi, J. Normand, K. Raghavachari, A. P. Rendell, J. C. Burant, S. S. Iyengar, J. Tomasi, M. Cossi, J. M. Millam, M. Klene, C. Adamo, R. Cammi, J. W. Ochterski, R. L. Martin, K. Morokuma, O. Farkas, J. B. Foresman, and D. J. Fox, Gaussian, Inc., Wallingford CT, **2016**.

[S7] U. Salzner, A. Aydin, *J. Chem. Theory Comput.* **2011**, *7*, 2568.

[S8] P. Murto, S. Elmas, U. A. Méndez-Romero, Y. Yin, Z. Genene, M. Mone, G. G. Andersson, M. R. Andersson, E. Wang, *Macromolecules* **2020**, *53*, 11106.

[S9] VMD 1.9.3 program: http://www.ks.uiuc.edu/Research/vmd/ .

VI. Copies of ^{1}H NMR, MALTI-TOF Mass spectra and GPC elution curves of P1-P5

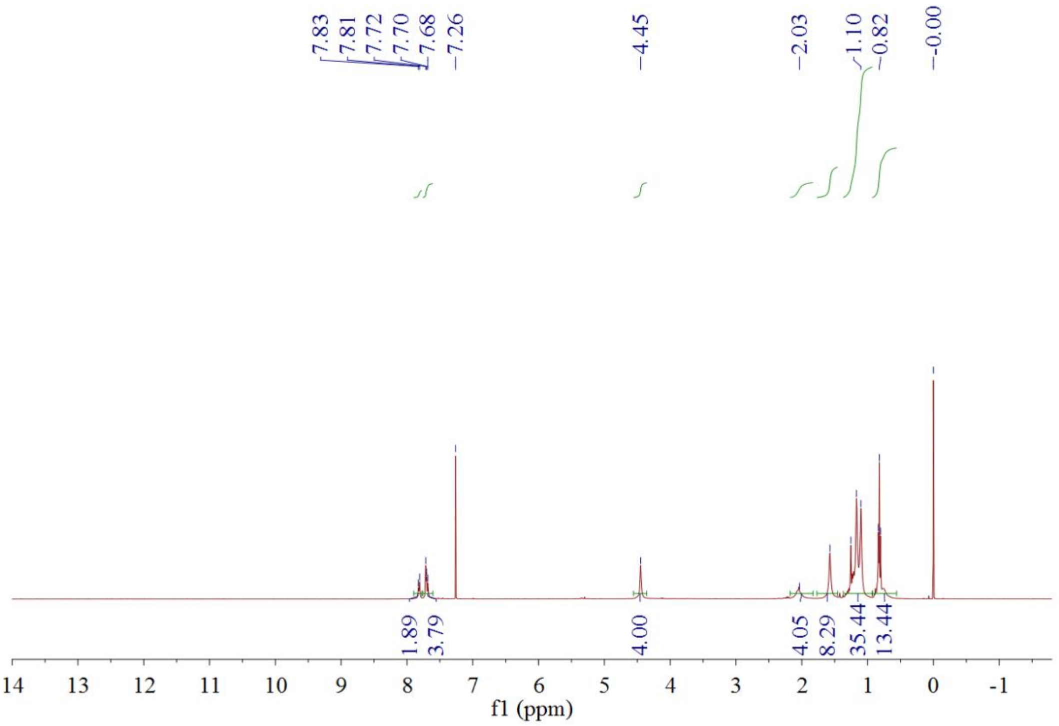

Figure S21 ^{1}H NMR spectrum of **P1** in $CDCl_3$.

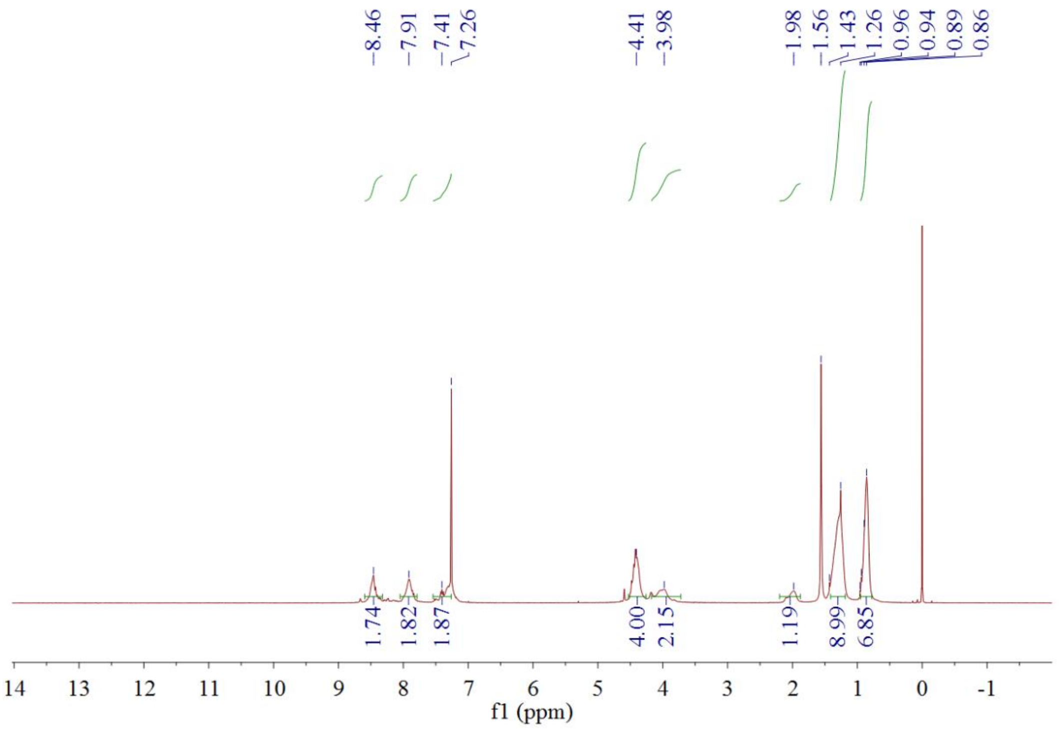

Figure S22 ^{1}H NMR spectrum of **P2** in $CDCl_3$.

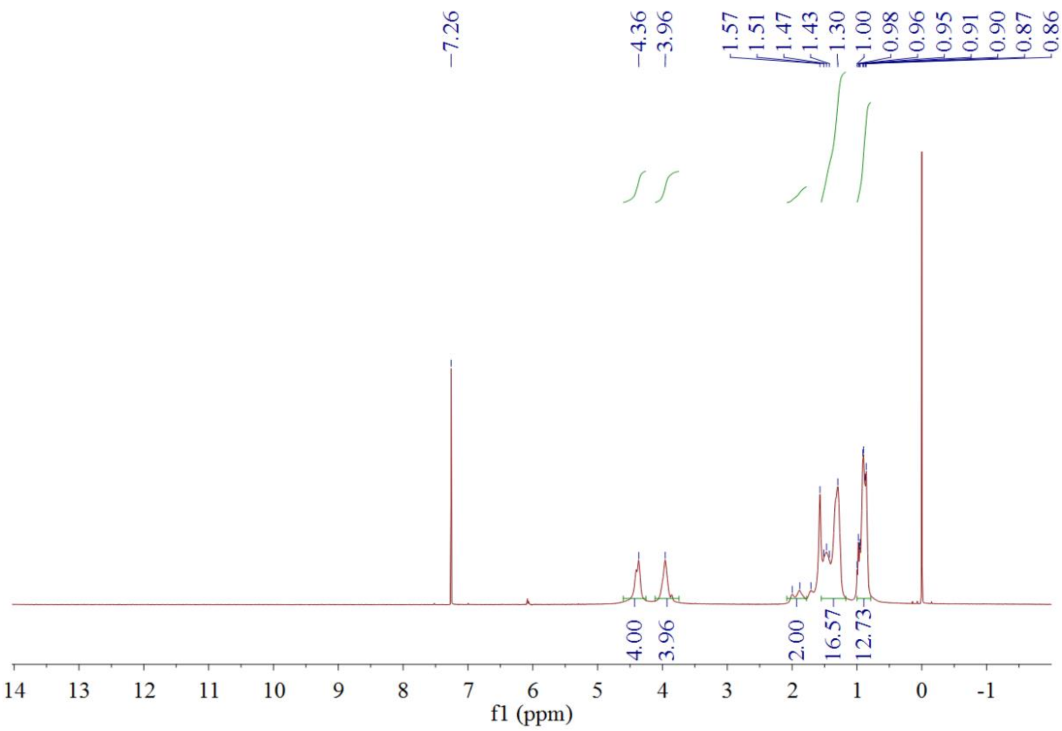

Figure S23 ^{1}H NMR spectrum of **P3** in $CDCl_3$.

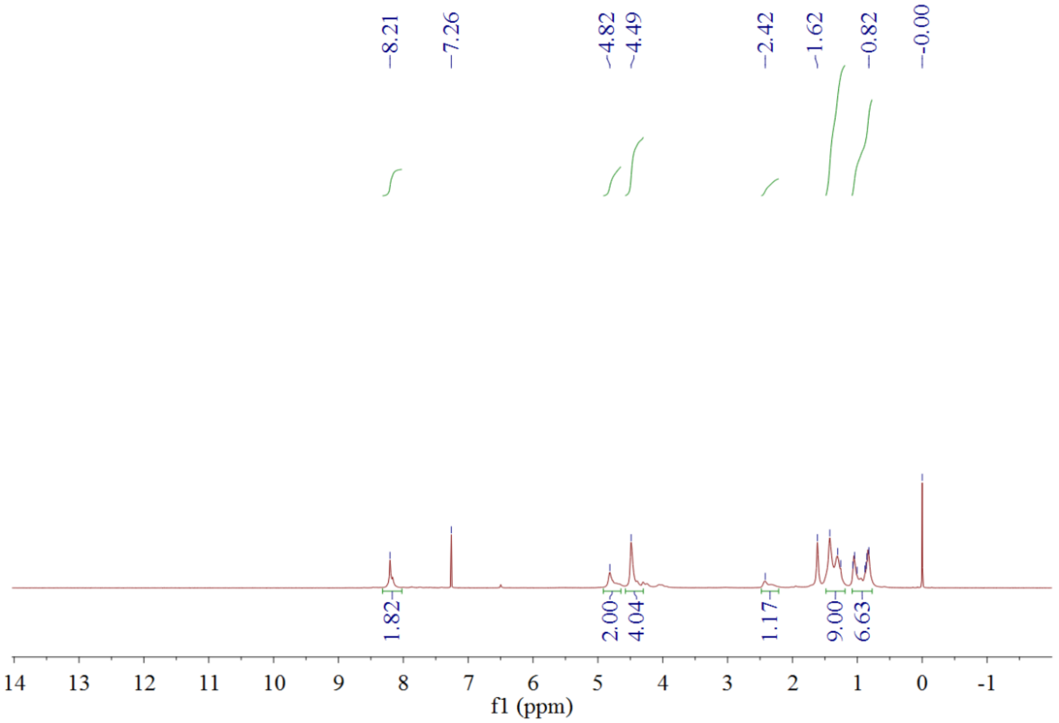

Figure S24 ^{1}H NMR spectrum of **P4** in $CDCl_3$.

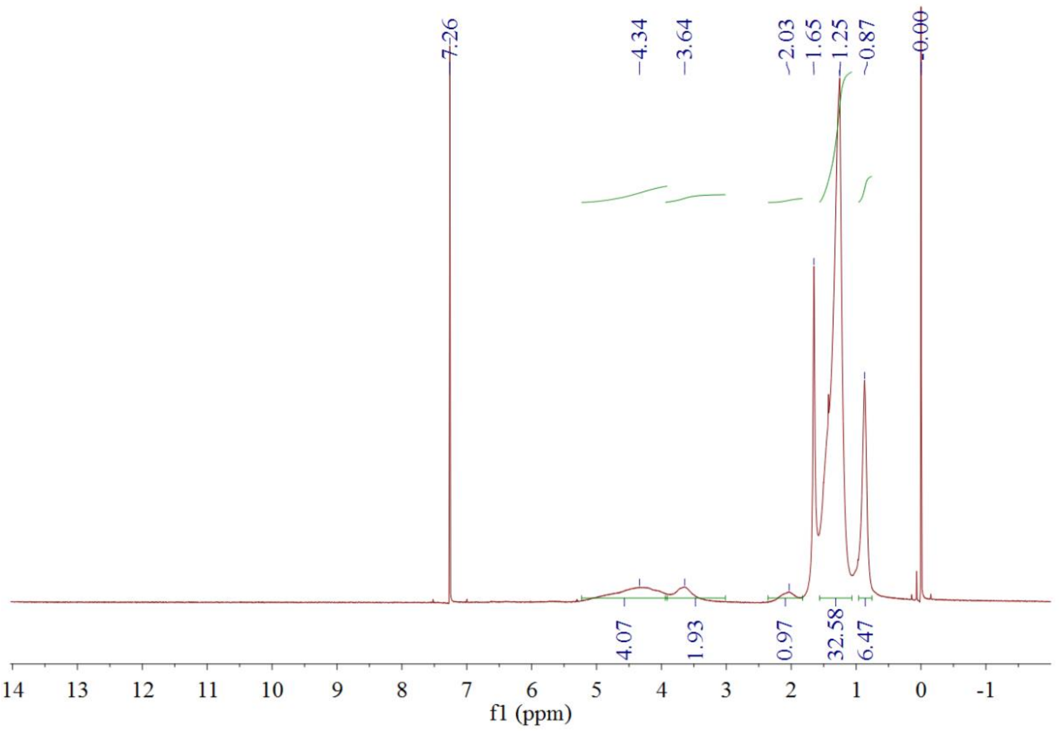

Figure S25 ^{1}H NMR spectrum of **P5** in $CDCl_3$.

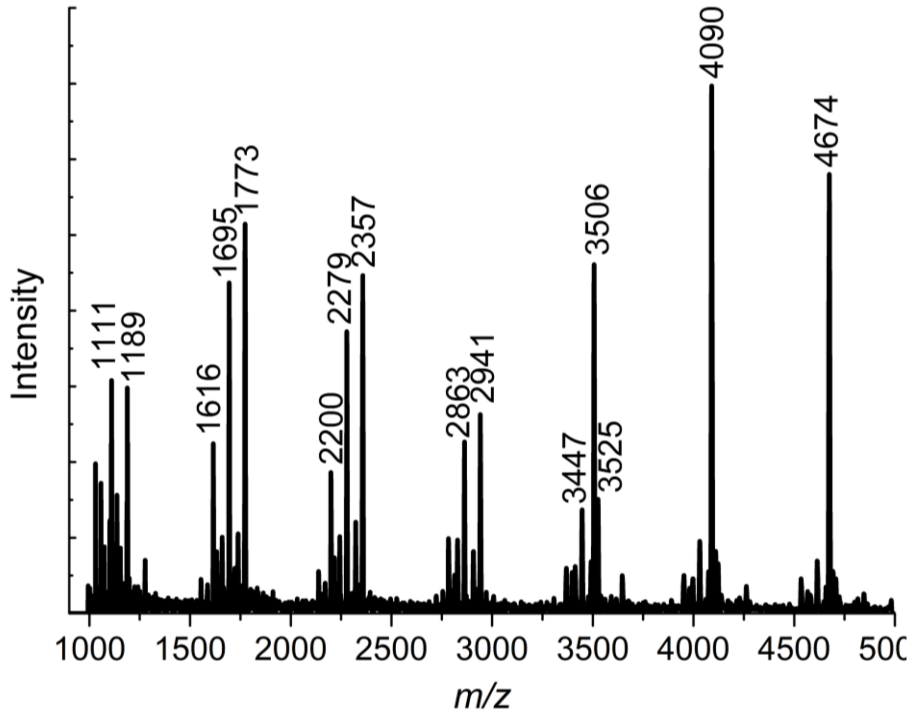

Figure S26 MALDI-TOF-MASS spectrum of **P1**, (calculated mass for repeating unit: 584).

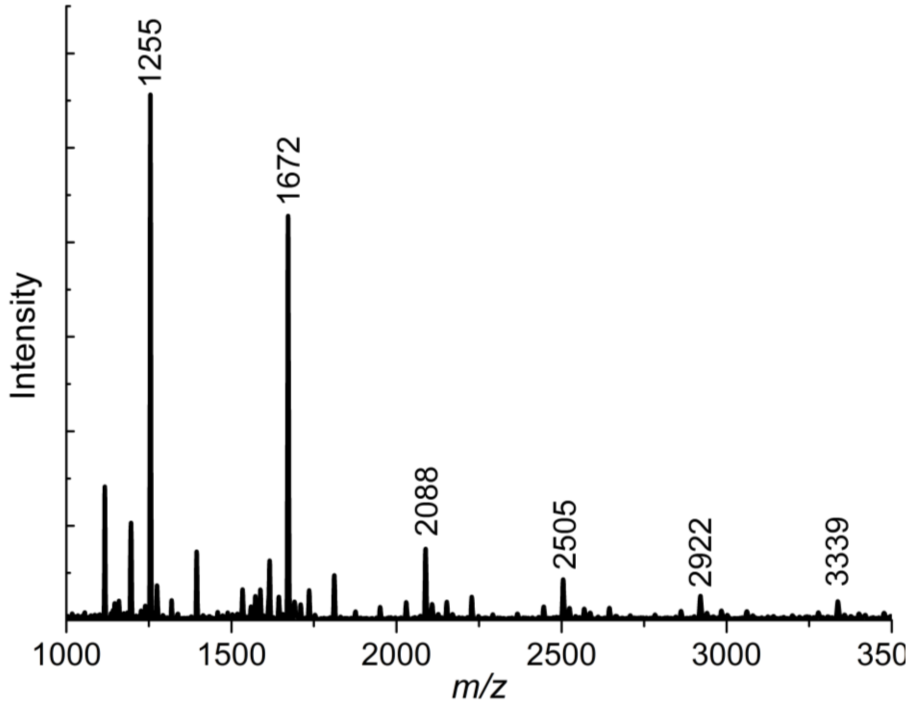

Figure S27 MALDI-TOF-MASS spectrum of **P2**, (calculated mass for repeating unit: 417).

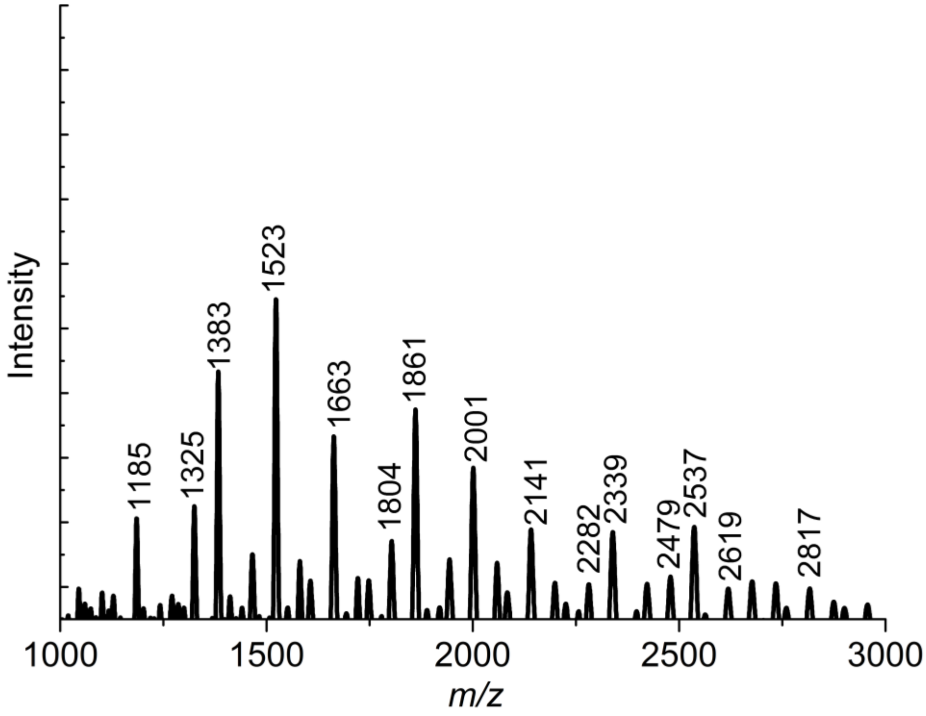

Figure S28 MALDI-TOF-MASS spectrum of **P3**, (calculated mass for repeating unit: 478).

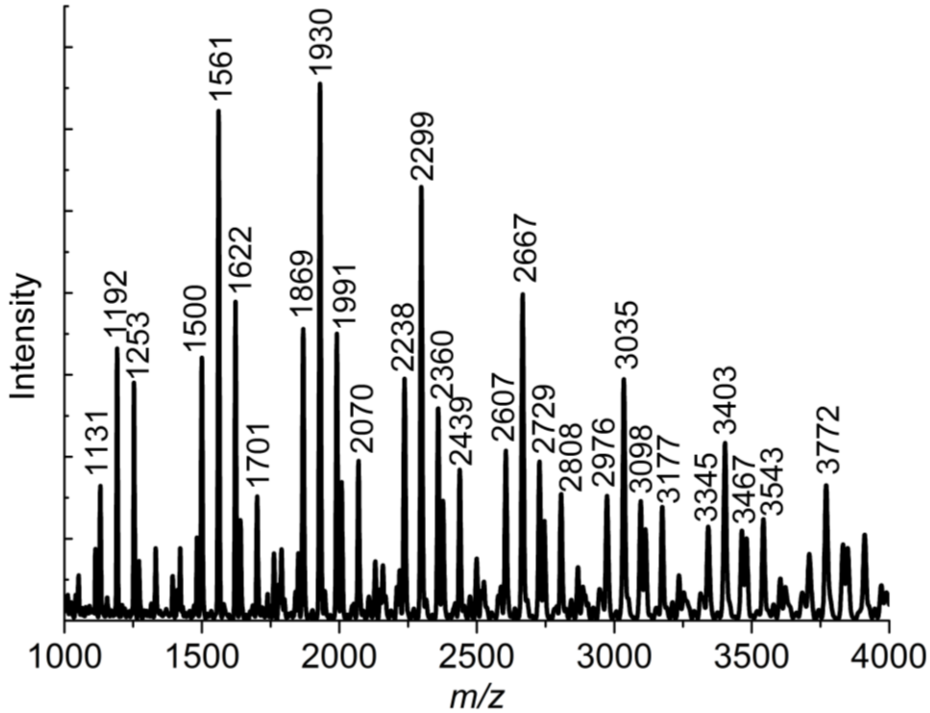

Figure S29 MALDI-TOF-MASS spectrum of **P4**, (calculated mass for repeating unit: 369).

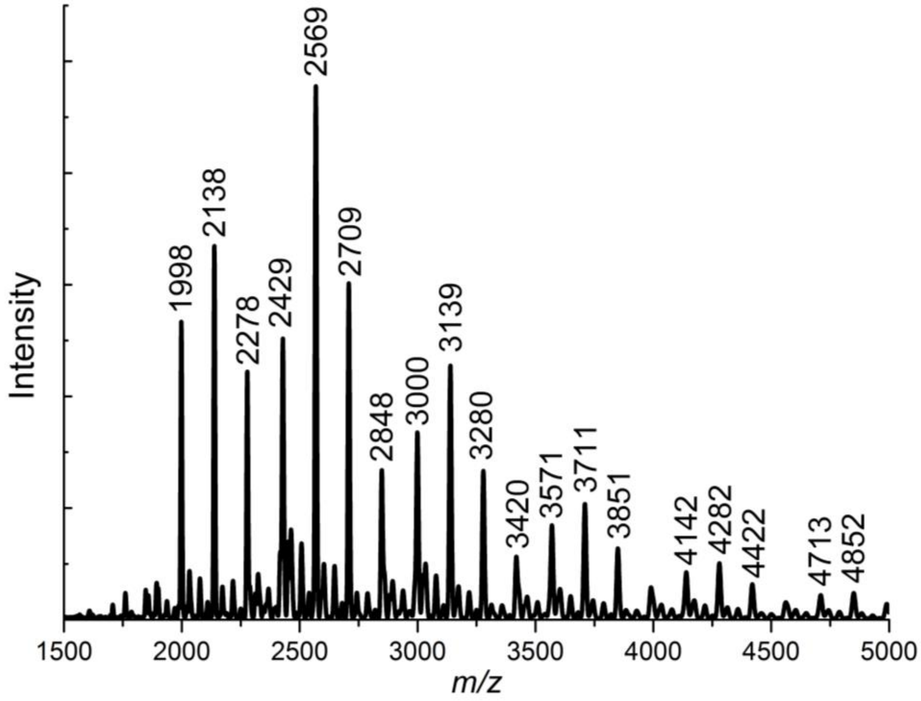

Figure S30 MALDI-TOF-MASS spectrum of **P5**, (calculated mass for repeating unit: 571).

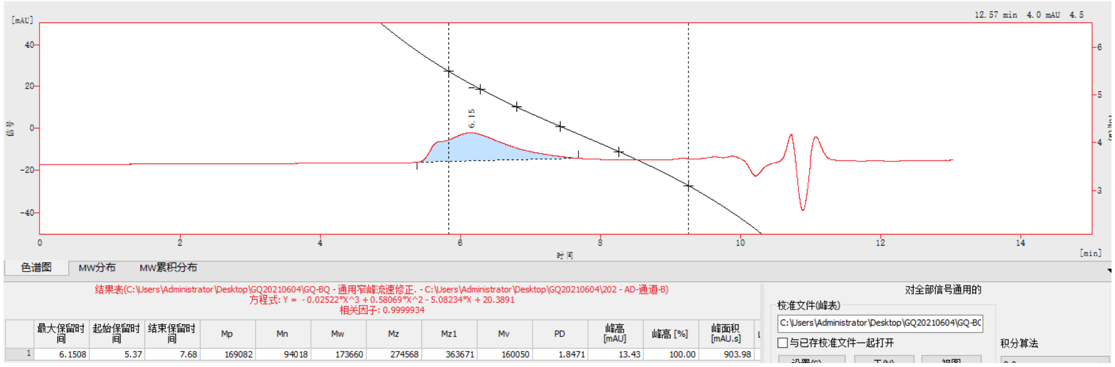

Figure S31 Copy of GPC elution curves and molecular weight data of **P1**.

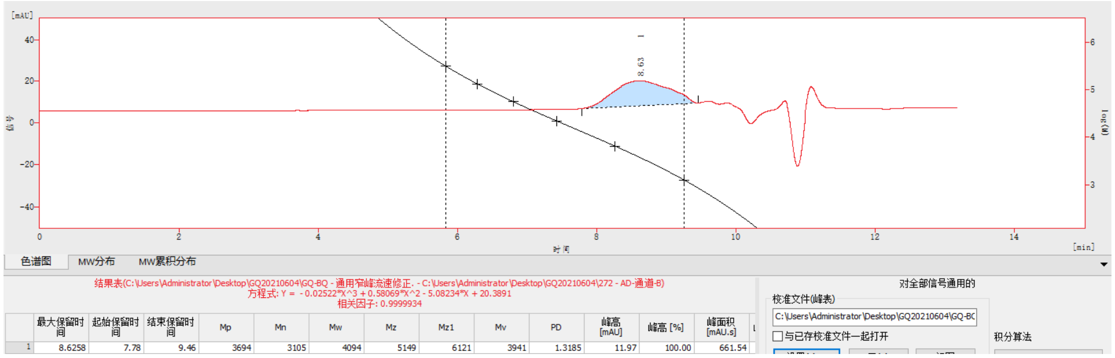

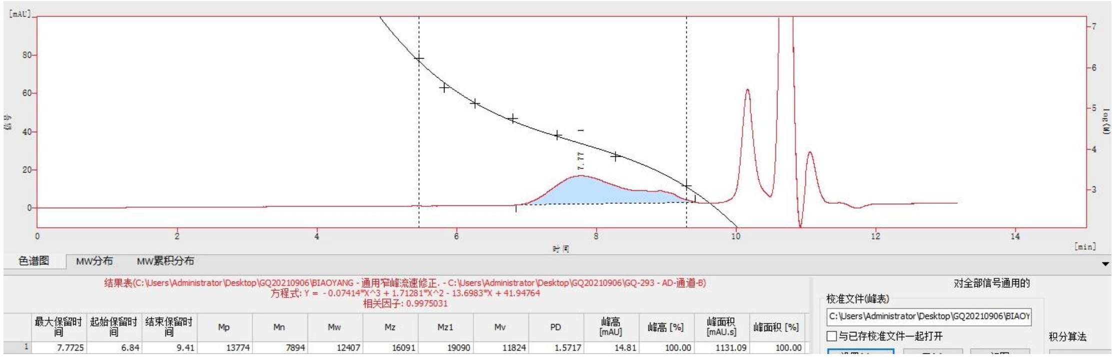

Figure S32 Copies of GPC elution curves and molecular weight data of **P2** obtained after 24h (upper) and 48 h (lower)

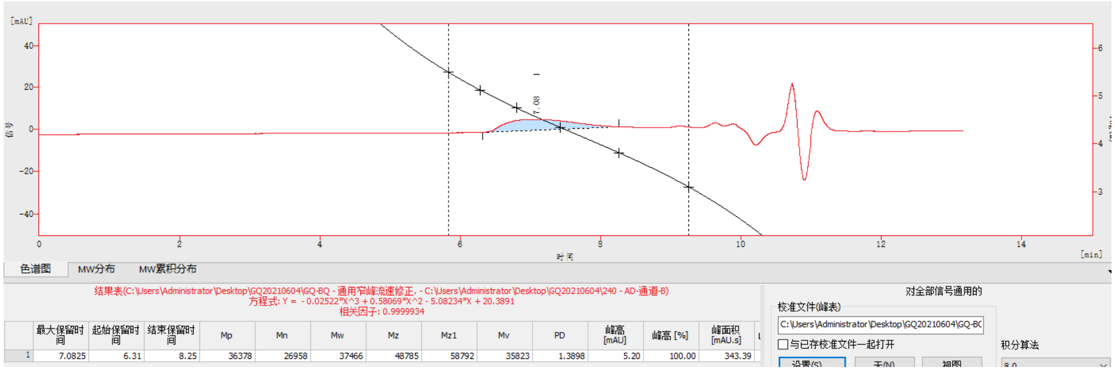

Figure S33 Copy of GPC elution curves and molecular weight data of **P3**

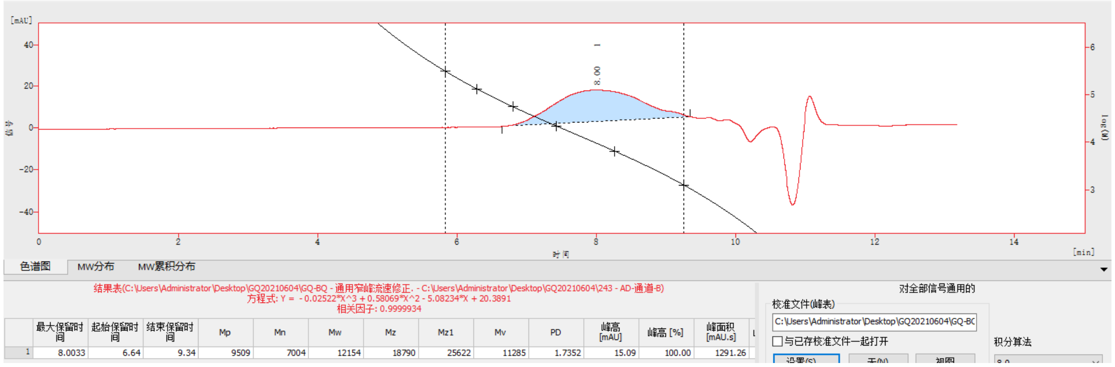

Figure S34 Copy of GPC elution curves and molecular weight data of **P4**

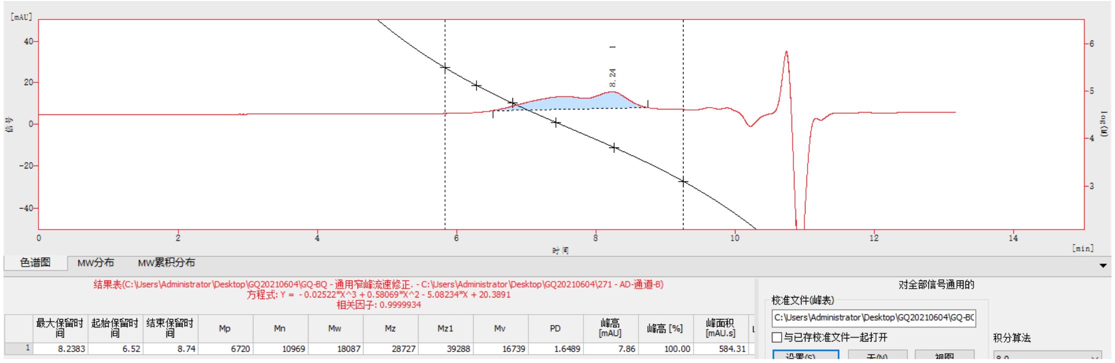

Figure S35 Copy of GPC elution curves and molecular weight data of **P5**.
